# An Integrated Approach to Characterize Intestinal Metabolites of Four Phenylethanoid Glycosides and Intestinal Microbe-Mediated Antioxidant Activity Evaluation *In Vitro* Using UHPLC-Q-Exactive High-Resolution Mass Spectrometry and a 1,1-Diphenyl-2-picrylhydrazyl-Based Assay

**DOI:** 10.3389/fphar.2019.00826

**Published:** 2019-07-25

**Authors:** Xiaoming Wang, Xiaoyan Chang, Xiaomei Luo, Meifeng Su, Rong Xu, Jun Chen, Yi Ding, Yue Shi

**Affiliations:** ^1^Institute of Medicinal Plant Development, Chinese Academy of Medical Science and Peking Union Medical College, Beijing, China; ^2^Beijing University of Chinese Medicine, Beijing, China; ^3^School of Pharmaceutical Sciences, Tsinghua University, Beijing, China

**Keywords:** intestinal microbial metabolism, four phenylethanoid glycosides, UHPLC-Q-Exactive Orbitrap HRMS, antioxidant activities, structure–activity relationship

## Abstract

Intestinal bacteria have a significant role in metabolism and the pharmacologic actions of traditional Chinese medicine active ingredients. Phenylethanoid glycosides (PhGs), as typical phenolic natural products, possess wide bioactivities, but low oral bioavailability. The aim of this work was to elucidate the metabolic mechanism underlying PhGs in the intestinal tract and screen for more active metabolites. In this study, a rapid and reliable method using an effective post-acquisition approach based on advanced ultra-high-performance liquid chromatography (UHPLC) coupled with hybrid Quadrupole-Orbitrap high resolution mass spectrometry (Q-Exactive-HRMS) provided full MS and HCD MS^2^ data. Thermo Scientific™ Compound Discoverer™ software with a Fragment Ion Search (FISh) function in one single workflow was developed to investigate the intestinal microbial metabolism of four typical PhGs. Furthermore, antioxidant activity evaluation of PhGs and their related metabolites was simultaneously carried out in combination with a 1,1-diphenyl-2-picrylhydrazyl (DPPH) assay to understand how intestinal microbiota transformations modulate biological activity and explore structure–activity relationships (SARs). As a result, 26 metabolites of poliumoside, 42 metabolites of echinacoside, 42 metabolites of tubuloside, and 46 metabolites of 2′-acetylacteoside were identified. Degradation, reduction, hydroxylation, acetylation, hydration, methylation, and sulfate conjugation were the major metabolic pathways of PhGs. Furthermore, the degraded metabolites with better bioavailability had potent antioxidant activity that could be attributed to the phenolic hydroxyl groups. These findings may enhance our understanding of the metabolism, pharmacologic actions, and real active forms of PhGs.

## Introduction

Recently, more and more attention has focused on the nutraceutical industry and preventive medications in the quest for natural antioxidants from plant materials. Polyphenolic compounds are well known to possess various pharmacologic effects, especially antioxidant activity (Mikulic-Petkovsek et al., 2016; Zhao et al., 2017; Kschonsek et al., 2018). Phenylethanoid glycosides (PhGs) are a class of polyphenolic glycoside compounds that are widely distributed in many plants. To date, various PhGs have been isolated and identified (Song et al., 2016a; Song et al., 2016b; Zou et al., 2017) In recent decades, pharmacologic studies have shown that the bioactivities of PhGs are diverse, including neuroprotective, cardioactive, hepatocyte protective, antioxidative, anti-inflammation, and immunomodulatory effects (Wang et al., 2012; Li et al., 2016c; Gu et al., 2016; Xue and Yang, 2016; Fu et al., 2018). These outstanding PhG activities in diverse diseases have proven importance in medicinal chemistry research.

Most herbal medicines are administered orally, and the components are inevitably metabolized before absorption from the gastrointestinal tract; however, poor oral absorption of PhGs was observed in a Caco-2 cell monolayer model, suggesting poor intestinal permeability (Gao et al., 2015; Zhou et al., 2018). A low blood drug concentration and relatively rapid metabolism of PhGs were observed after dosing in previous pharmacokinetic studies (Qi et al., 2013; Deng et al., 2014; Li et al., 2015a; Cui et al., 2016a; Li et al., 2016a; Su et al., 2016; Cui et al., 2017; Feng et al., 2018; Qian et al., 2018); however, these existing results are inadequate to fully understand the metabolic process and the mechanism underlying the pharmacologic activities. An *in vitro* digestion model provides a useful platform for fast and reproducible assessment of herbal medicine metabolism (Payne et al., 2012; Kang et al., 2013; Xu et al., 2017; Cui et al., 2018; Zhang et al., 2018; Feng et al., 2019). Several *in vitro* studies involving the metabolism of PhGs (echinacoside, acteoside, isoacteoside, and 2′-acetylacteoside) showed that PhGs are easily transformed into degradation products, such as caffeic acid (CA) and hydroxytyrosol (HT), by human or rat intestinal bacteria or intestinal enzymes (Li et al., 2015b; Cui et al., 2016b; Li et al., 2016b; Li et al., 2017). These degradation products are involved in further metabolism, which is reasonable for the low oral bioavailability of PhGs. These results also suggested that PhGs may serve as a prodrug and is transformed by intestinal flora leading to the occurrence of new metabolites with increased activity. Consequently, more attention should be given to these metabolites and the biological properties to help explain the health effects of PhGs that are not easily absorbed through the gut barrier.

In the current study, we systematically characterized the metabolic process of four typical PhGs (echinacoside, poliumoside, 2′-acetylacteoside, and tubuloside A) using a UHPLC-HRMS Orbitrap™ instrument with high specificity, sensitivity, and accuracy. A full scan with data-dependent MS^2^ (ddMS^2^) acquisition is enabled in one run, providing complementary information for the structural elucidation of metabolites. Compound Discoverer software is the only small-molecule analysis solution able to make full use of the rich high-resolution accurate-mass (HRAM) data produced by Orbitrap mass spectrometers. The application of LC-HRAM with post-acquisition data processing using new Compound Discoverer software was verified in accurate and batch metabolite identification. Transformation sites and pathways of the four components were also proposed. Although we found that these PhGs are extensively metabolized by intestinal bacteria, little is known about the role of intestinal bacteria in bioactivity modification of PhG types. Accordingly, these PhGs and related metabolites should be simultaneously taken into account when assessing bioactivities. Previous studies have shown that PhGs as powerful natural antioxidants relevant to the phenolic group and some of the intestinal microbial metabolites still bear a phenolic group and have been suggested to possess strong antioxidant activities (Dueñas et al., 2015; Jiang et al., 2016; Ydjedd et al., 2017; Wang et al., 2017a). In this regard, the antioxidant activities were compared with the 1,1-diphenyl-2-picrylhydrazyl (DPPH)-based assay *in vitro* for the first time. Some selected metabolites have been used to carry out structure–activity relationship (SAR) studies to better understand which structural features are essential for biological activity. Based on the SARs, this is also the first report to elucidate these intestinal microbe-mediated biotransformation modulations on antioxidant activities *in vitro* to date. Hence, our results may provide helpful information to better understand *in vivo* metabolism, mechanism of action of PhGs, and drug discovery in clinical development research.

## Materials and Methods

### Chemicals, Reagents, and Animals

**Figure 1:**
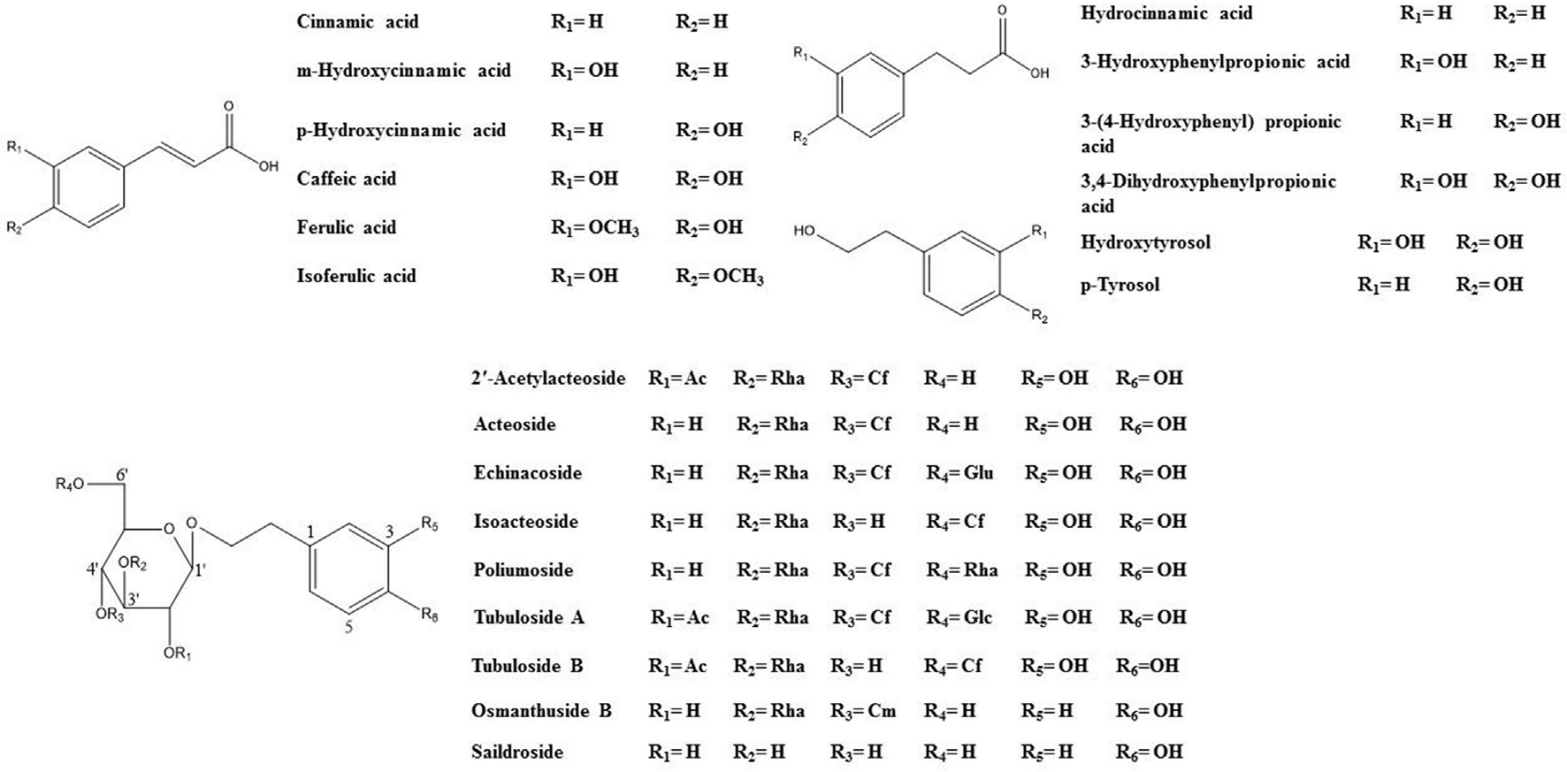
Chemical structures of reference standards used in this study.

Reference standards for echinacoside, poliumoside, 2′-acetylacteoside, tubuloside A, acteoside, isoacteoside, saildroside, HT, and CA were purchased from Mansite Bio-Technology Co., Ltd. (Chengdu, China). Tubuloside B and Osmanthuside B were purchased from Chroma-Biotechnology Co., Ltd. (Chengdu, China) and 3,4-dihydroxybenzenepropanoic acid, 3-hydroxyphenylpropionic acid (3-HPP), 3-(4-hydroxyphenyl) propionic acid, hydrocinnamic acid, cinnamic acid, p-hydroxycinnamic acid, m-hydroxycinnamic acid, ferulic acid, isoferulic acid, and p-tyrosol were purchased from Herbpurfty Co., Ltd. (Chengdu, China). The structures are shown in **Figure 1** and the purity of each reference standard was determined to be >98% by normalization of the peak areas detected by HPLC-DAD-TOF/MS and the reports of 13 C-NMR analysis. DPPH, L-ascorbic acid, and trolox were purchased from Sigma-Aldrich (St. Louis, MO, USA). The solvents, acetonitrile and methanol, were of HPLC grade and obtained from Merck (Darmstadt, Germany). Formic acid (MS grade) was purchased from Fisher Scientific (Madrid, Spain). Deionized water (18 MΩ) was prepared by distilled water through a Milli-Q system (Millipore, Bedford, MA, USA). All of the other chemicals and reagents were of analytical grade. AnaeroPack rectangular jars were purchased from Mitsubishi Gas Chemical Company, Inc. (Tokyo, Japan). General anaerobic medium (GAM) was purchased from Shanghai Kayon Biological Technology Co., Ltd. (Shanghai, China). Corning (Costar) Inc. (Tewksbury, MA, USA) was the vendor for 96-well plates.

Ten male Sprague–Dawley rats (7–8 weeks old and weighing 220 ± 20 g) were obtained from Weitonglihua Company (Beijing, China) and housed with free access to standard food and water. The animals were kept under controlled conditions (temperature, 22–24°C; relative humidity, 50 ± 10%) with a 12-h light/12-h dark cycle and acclimated to the housing environment for 1 week prior to the experiment with free access to food and distilled water. All of the experiments were performed according to the National Institutes of Health Guidelines for Animal Research and approved by the Ethics Committee of the Institute of Medicinal Plant Development, CAMS & PUMC.

### Incubation of Four PhGs With Rat Intestinal Bacteria

GAM content (g/L) was as follows: 15.0 g of peptone; 10.0 g of tryptone; 3.0 g of soya peptone; 5.0 g of yeast extract; 13.5 g of digestibility serum powder; 1.2 g of beef extract; 3.0 g of glucose; 2.5 g of KH_2_PO_4_; 3.0 g of NaCl; 0.3 g of soluble starch; 0.3 g of L-cysteine hydrochloride; 0.15 g of sodium thioglycolate; and 6.0 g of anaerobic broth powder. Thirty grams of GAM was dissolved in 1,000 mL of distilled water, filtered while hot, treated with an anti-bacterial process with high pressure (0.15 MPa) and temperature (121°C) for 20 min, and cooled to 45°C, and 1 mg of vitamin K1 and 6 mg of hematin chloride were dissolved in the GAM broth solution.

Fresh fecal samples were obtained from 10 male Sprague–Dawley rats. The fresh rat feces were mixed immediately and homogenized with 4 volumes of aseptic physiologic saline using a vortex mixer. The suspension was centrifuged at 2,000 rpm for 10 min, and the suspension was inoculated into 9 volumes of GAM broth, which was then incubated at 37°C in an anaerobic pack for 24 h. The resulting mixture of bacteria was centrifuged at 4000 rpm for 10 min, and the residue was suspended in aseptic physiologic saline to be used as the intestinal bacterial mixture.

One milliliter of GAM broth containing 0.1 mL of intestinal bacterial mixture and the 4 PhGs (1 mM) were separately transferred to 2-mL Eppendorf tubes and incubated at 37°C for 24 h in anaerobic cultivation bags sealed with anaerobic airbags. Incubation of intestinal bacteria in medium, but lacking the PhG solutions, was used to monitor metabolites arising from basal metabolism (control groups).

### Sample Preparation

The cultured mixture was removed and extracted with water-saturated n-butanol (1:1, v/v) after incubation for 24 h. The mixture was centrifuged at 4,800 rpm for 20 min and then the supernatant was concentrated under a stream of nitrogen at room temperature. The residue was dissolved in 0.5 mL of methanol and centrifuged at 12,000 rpm for 10 min and then the supernatant was analyzed by UHPLC-Q-Exactive Orbitrap HRMS.

### UHPLC-Q-Exactive Orbitrap HRMS Analysis Condition

Ultra-high-performance liquid chromatography (UHPLC) analyses were performed using an Ultimate 3000 system (Dionex, Sunnyvale, CA, USA) that was equipped with an online vacuum degasser, a quaternary pump, an autosampler, and a column compartment with a thermostat. The column that was used for the chromatographic separation was a Waters Acquity UPLC HSS T3 (1.7 μm, 2.1 mm ×100 mm; Waters, Milford, MA, USA) at 35°C. The separation conditions consisted of a gradient elution using acetonitrile as mobile phase A and aqueous formic acid 0.1% (v/v) as phase B at a flow rate of 0.3 mL/min. The following gradient was applied: 0–8 min, 2%–12% A; 8–16 min, 12–16% A; 16–19 min, 16% A; 19–22 min, 16%–19% A; 22–25 min, 19% A; 25–30 min, 19%–25% A; 30–32 min, 25% A; 32–35 min, 25%–30% A; 35–38 min, 30%–40% A; 38–40 min, 40%–50% A. The injection volume was 2 µL and the injection temperature was 15°C.

Tandem mass spectrometry was performed with a Q Exactive Orbitrap MS (Thermo Fisher, Waltham, MA, USA) using a heated electrospray ionization source for the ionization of the target compounds in the negative ion (NI) mode. The operating parameters were as follows: spray voltage, 2.00 kV; sheath gas pressure, 30 psi; auxiliary gas pressure, 10 arb; capillary temperature, 320°C; auxiliary gas heater temperature, 350°C; scan modes, full MS (resolution 70,000) and ddMS^2^ [resolution 17,500, with stepped collision energy (20, 40, and 60 eV)]; and scan range, *m*/*z* 80–1200. All data were acquired using the Xcalibur 3.1 software (Thermo Scientific).

### DPPH Radical Scavenging Assay

The antioxidant activity of PhG and some metabolite-related analogs was assessed using a feasible and rapid DPPH radical scavenging assay *in vitro*. Each sample stock solution was diluted to final concentrations of 0.05–2,000 μg/mL in MeOH. The DPPH radical standard solution (1 × 10^−4^ mol/L) was prepared fresh by immediately dissolving an accurately weighed DPPH sample in ethanol while protected from light on the day of each test. One milliliter of each sample solution at various concentrations was mixed with 1 mL of 1 × 10^−4^ mol/L DPPH solution (in ethanol). All samples were shaken and allowed to stand in the dark at room temperature for 30 min. The reduction in DPPH-free radicals was measured by reading the absorbance at 517 nm against a blank on a microplate reader (Tecan, Switzerland). One milliliter of ethanol plus 1 mL of sample solution was used as a blank, while 1 mL of DPPH solution plus 1 mL of MeOH was used as a negative control. Trolox and L-ascorbic acid were used as positive controls. The radical scavenging activity (% inhibition) of the tested samples was expressed as the DPPH scavenging percentage and calculated using the following formula: % inhibition = [(A control − A sample)/A control] × 100. The antioxidant activity was calculated by plotting the percent inhibition against the sample concentration and represented as the sample concentration required to scavenge 50% of the DPPH radical (IC_50_). All tests were carried out in triplicate, and the IC_50_ values were reported as the mean ± SD.

### Data Processing

The raw MS data files of the blank matrix and the control and sample groups were imported into Compound Discoverer™ software (v.2.1; Thermo Scientific, Fremont, CA, USA) to identify metabolites of the four PhGs. Compound Discoverer^TM^ software can quickly find and identify metabolites with background removal from the blank matrix. A list of potential metabolites was generated depending on the HRAM measurement, as follows: each within ±5 ppm of mass error; retention time tolerance of ±0.1 min; an ion ratio tolerance within ±30%; and fine isotopic pattern matching >90% of the precursor and the characteristic product ions. Furthermore, structural elucidations and transformations were suggested for each chromatographic peak by the Fragment Ion Search (FISh) function in Compound Discoverer^TM^ software. The FISh coverage score was calculated and fragments are auto-annotated with structure, molecular weight, and elemental composition on MS/MS spectra. The expected compounds table was filtered by background and maximum area ≥ 10^5^ or FISh coverage score ≥ 50.

## Results and Discussion

### Fragmentation Studies

**Figure 2:**
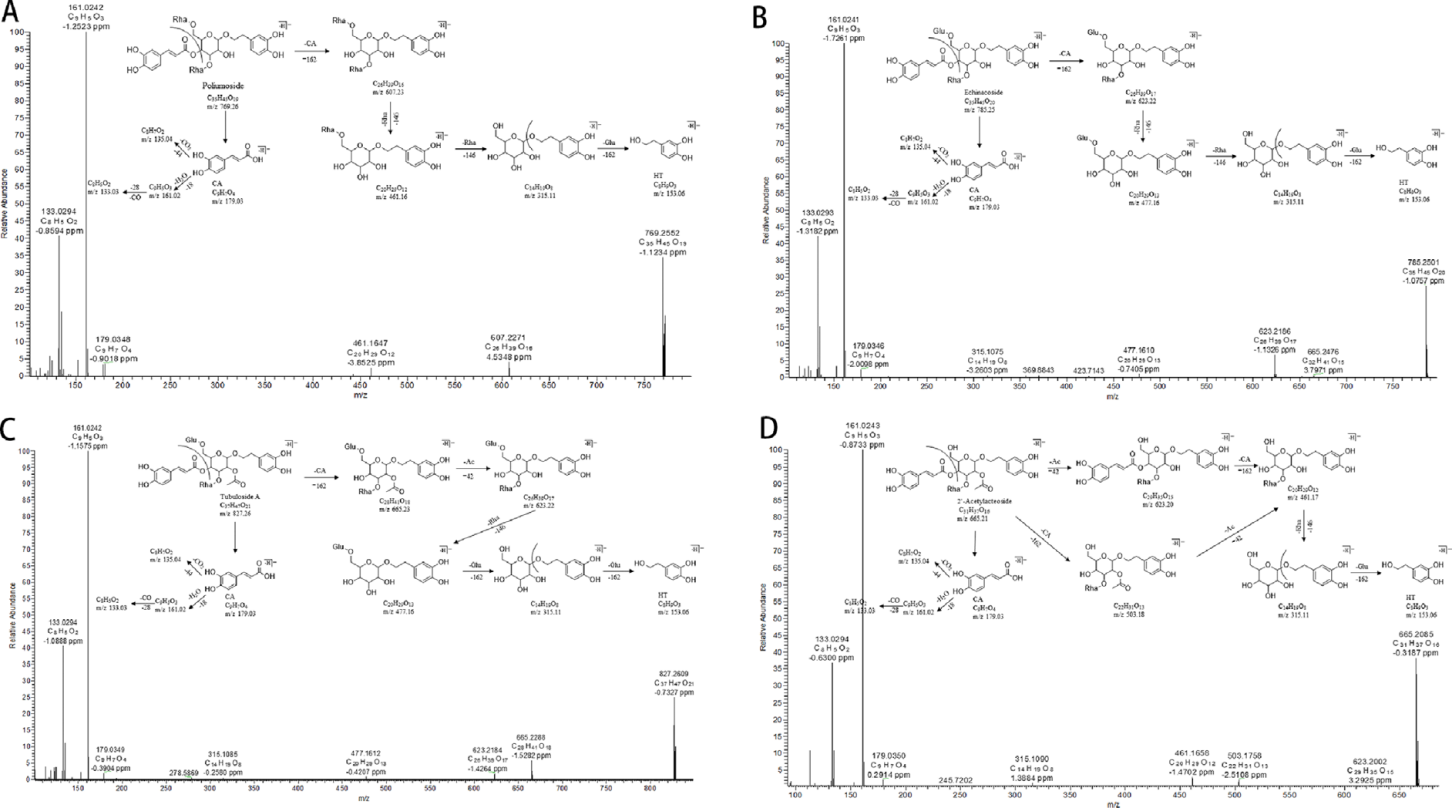
UHPLC-MS/MS spectra and proposed fragmentation pathways of poliumoside **(A)**, echinacoside **(B)**, tubuloside A **(C)**, and 2′-acetylacteoside **(D)**.

**Figure 3:**
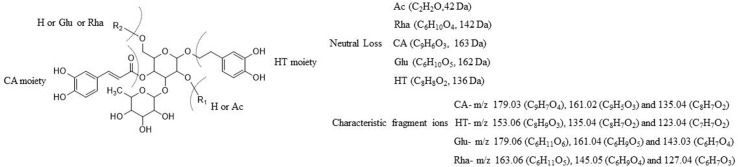
The common fragmentation patterns of the four PhGs.

As reported, the common chemical structure of PhGs is characterized by a phenethyl alcohol (C6-C2) moiety, such as HT or p-tyrosol, attached to a β-glucopyranose/β-allopyranose *via* a glycosidic bond, side-chain aromatic acids (e.g., CA or coumaric acid), and sugar groups, including rhamnose (Rha) and glucose (Glu) (Jiang and Tu, 2009; Han et al., 2012; Zheng et al., 2014). In this work, eight reference standards were used for the fragmentation patterns study using UHPLC-Q-Exactive Orbitrap HRMS, which is helpful in further metabolite characterization. The detailed fragmentation patterns of echinacoside, acteoside, isoacteoside, 2′-acetylacteoside, and tubuloside B have been reported in our previous study by UPLC-ESI-QTOF/MS^n^ (Wang et al., 2017b). In the NI mode of Q-Exactive Orbitrap HRMS, the fragmentation patterns of other parent compounds, such as poliumoside and tubuloside A, were first proposed in our study. By way of comparison with echinacoside, poliumoside is also a trisaccharide glycoside; however, there is a Rha, not a Glu unit, at the C-6′ position of the central Glc unit. Therefore, the other fragmentation patterns of poliumoside were similar to those of echinacoside, apart from the different neutral loss of the Rha or Glu moiety. Tubuloside A is also a trisaccharide glycoside. The structure of tubuloside A has an additional Glc group conjugated at the C-6′ position of the central Glc unit, and when compared with 2′-acetylacteoside, the fragmentation of tubuloside A can also be predicted. The proposed fragment ion structures and stepwise elucidations on the fragmentation patterns are illustrated in **Figure 2**. In the present study, the common fragmentation patterns (**Figure 3**) with the MS^n^ data in the NI mode were systematically summarized as follows: 1) the major and typical neutral losses corresponding to the loss of Ac (C_2_H_2_O, −42 Da), CA (C_9_H_6_O_3_, −162 Da), HT (C_8_H_8_O_2_, −136 Da), Rha (C_6_H_10_O_4_, −146 Da), and Glu (C_6_H_10_O_5_, −162 Da) moieties; 2) a series of the lower-molecular-weight characteristic fragment ions also possess significant regularities corresponding to three types of substituent moieties, such as the CA moiety (*m*/*z* 179.03 [C_9_H_7_O_4_]) that produced ions at *m*/*z* 135.04 (C_8_H_7_O_2_) and 161.02 (C_9_H_5_O_3_) by the further loss of CO_2_ and H_2_O, respectively; 3) the HT moiety (*m*/*z* 153.06 [C_8_H_9_O_3_]) that produced ions at *m*/*z* 123.04 (C_7_H_7_O_2_) and 135.04 (C_8_H_7_O_2_) by the further loss of CH_2_O and H_2_O, respectively; and 4) the Rha moiety (*m*/*z* 163.06 [C_6_H_11_O_5_]) along with ions at *m*/*z* 145.05 (C_6_H_9_O_4_) and 127.04 (C_6_H_7_O_3_) or the Glu moiety (*m*/*z* 179.06 [C_6_H_11_O_6_]), along with ions at *m*/*z* 161.04 (C_6_H_9_O_5_) and 143.03 (C_6_H_7_O_4_), both of which are produced by the successive loss of H_2_O.

For other phenolic compounds, 3,4-dihydroxybenzenepropionic acid [*m*/*z* 181.05 (C_9_H_9_O_4_)] produced fragment ions at *m*/*z* 137.06 (C_8_H_9_O_2_) by loss of CO_2_, which then produced ions at *m*/*z* 119.05 (C_8_H_7_O) *via* loss of H_2_O. CO_2_ was lost from 3-hydroxyphenylpropionic [*m*/*z* 165.06 (C_9_H_9_O_3_)] to form the ion at *m*/*z* 121.06 (C_8_H_9_O), and p-hydroxycinnamic acid [*m*/*z* 163.04 (C_9_H_7_O_3_)] produced fragment ions at *m*/*z* 145.03 (C_9_H_5_O_2_) and 119.05 (C_8_H_7_O) by further loss of H_2_O and CO_2_, respectively. Similarly, CO_2_ and H_2_O were also lost from CA [*m*/*z* 179.03 (C_9_H_7_O_4_)] to form the ion at *m*/*z* 135.04 (C_8_H_7_O_2_) and 161.02 (C_9_H_5_O_3_), respectively. CH_2_O and H_2_O were also lost from HT [*m*/*z* 153.06 (C_8_H_9_O_3_)] to form the ions at *m*/*z* 123.04 (C_7_H_7_O_2_) and 135.04 (C_8_H_7_O_2_), respectively, and p-tyrosol [*m*/*z* 137.06 (C_8_H_9_O_2_)] yielded product ions at *m*/*z* 107.05 (C_7_H_7_O) and 119.05 (C_8_H_7_O), which was due to the loss of CH_2_O and H_2_O, respectively. The proposed fragmentation patterns here would therefore be useful for further identifications of more PhGs and the related metabolites.

### Metabolite Identification of Four PhGs by Rat Intestinal Bacteria

**Figure 4:**
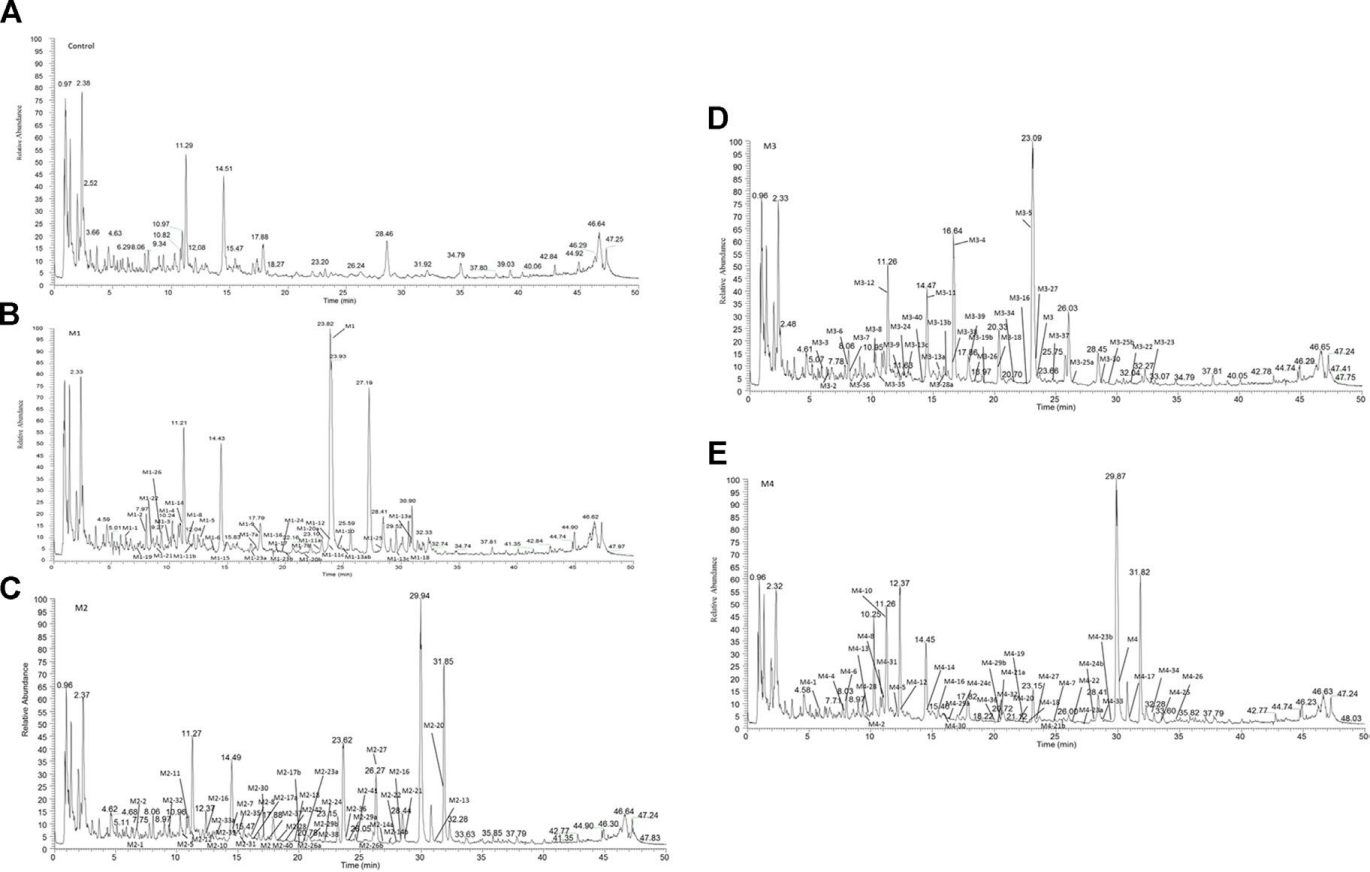
Total ion chromatograms of control **(A)**, M1 **(B)**, M2 **(C)**, M3 **(D)**, and M4 **(E)** incubated with rat intestinal bacteria for 24 h by UHPLC-Q-Exactive Orbitrap/MS.

In this study, UHPLC-Q-Exactive Orbitrap HRMS with Compound Discoverer software were used to investigate the metabolic profiles and identify the metabolites of four typical PhGs by rat intestinal bacteria. The total ion chromatograms are shown in **Figure 4**. The retention time, accurate mass of the quasi-molecular ion, and fragment ions for parent compounds (M1–M4) and all metabolites (M1-1-M1-26, M2-1-M2-42, M3-1-M3-42, and M4-1-M4-36) are listed in **Table S1**.

Metabolites were classified into the following three categories: 1) degradation products of parent compounds, 2) phase I and II metabolites of parent compounds, and 3) phase I and II metabolites of degradation products. The metabolite identification strategy and results are elucidated in detail, as follows.

#### Degradation Products of Parent Compounds

As described in the section Metabolite Identification of Four PhGs by Rat Intestinal Bacteria, parent compounds, including M1 [*m*/*z* 769.25592 (C_35_H_45_O_19_), eluted at 23.85 min], M2 [*m*/*z* 785.25031 (C_35_H_45_O_20_), eluted at 16.73 min], M3 [*m*/*z* 827.26190 (C_37_H_47_O_21_), 23.54 min], and M4 [*m*/*z* 665.20874 (C_31_H_37_O_16_), 29.91 min], were identified as poliumoside, echinacoside, tubuloside A, and 2′-acetylacteoside, respectively, by comparing the retention times and mass spectra with those of authentic reference standards (**Figure 2**). The fragment ion of the parent compound resulting from in-source by high energy collision dissociation (HCD) has exactly the same structure of one of the main degradation products because the glycosidic or ester bonds of the compound that can be hydrolyzed easily by intestinal bacteria in the alimentary tract are also the more likely to be broken during in-source fragmentation (**Figure 3**). Therefore, the degradation pathways, including de-rhamnose, deglycosylation, de-caffeoyl, de-HT, and deacetylation, are always similar to the mass fragmentation patterns. Moreover, the identification process of these compounds is simplified with automatic batch FISh annotations *via* direct fragment match.

Accordingly, we found that degradation products were formed by a combination of stepwise cleavage of Rha (146 Da), Glu (162Da), CA (162 Da), HT (136 Da), or Ac (42 Da) moieties *via* the “fragmentation–degradation” comparisons with the parent compound. Moreover, the detected diagnostic product ions originated from the following are also the fragment characteristic of the core chemical groups within the parent molecule: a) HT; b) CA; and c) Glu or Rha moieties. As discussed, all degradation products have been found to characterize the quasi-molecular ions with the same *m*/*z* and calculated elemental compositions to that of the parent compound fragments. The results are illustrated in detail as follows.

M1-20a or isomers [*m*/*z* 623.20 (C_29_H_35_O_15_), eluted at 23.14 and 26.04 min, respectively] were 146 Da (C_6_H_10_O_4_) less than M1 and the fragment ion at *m*/*z* 461.17 (C_20_H_29_O_12_) was formed by a loss of CA (C_9_H_6_O_3_, −162 Da) and yielded product ions associated with CA at *m*/*z* 179.03, 161.02, and 135.04. Fragment ions at *m*/*z* 153.06 and 123.04 were attributed to the HT group. M1-20a or isomers were assigned as C-6′ de-Rha products of M1 and confirmed as acteoside and isoacteoside, respectively, by comparison with reference standards. Thus, M1-20b or isomers [*m*/*z* 623.20 (C_29_H_35_O_15_), eluted at 22.22 and 25.14 min, respectively] were deduced as C-3’ de-Rha products of M1. M1-21 [*m*/*z* 487.14523 (C_21_H_27_O_13_), eluted at 8.86 min] was 136 Da (C_8_H_8_O_2_) less than M1-20 or isomers and also yielded product ions associated with CA at *m*/*z* 179.03, 161.02, and 135.04, suggesting that M1-21 was the de-Rha and de-HT product of M1. M1-2 [*m*/*z* 341.08850 (C_15_H_17_O_9_), eluted at 7.97 min] was 146 Da (C_6_H_10_O_4_) less than M1-21, and the diagnostic fragmentation from the CA moiety was found at *m*/*z* 179.03, 161.02, and 135.04, suggesting that M1-2 was the de-HT and de-2Rha product of M1. M1-3 eluted at 9.94 min and was characterized with the quasi-molecular ion at *m*/*z* 607.22443 (C_26_H_39_O_16_), which is really close to the de-CA fragment ion (*m*/*z* 607.22437) of M1 and is proposed to be the de-CA product of M1. The major fragment ions of M1-3 (*m*/*z* of 461.17 and 315.11) were also the same as from M1. The diagnostic fragmentation from the HT moiety was found at *m*/*z* 153.06 and 123.04. Based on this rule, M1-22 [*m*/*z* 461.16632 (C_20_H_29_O_12_), eluted at 8.07 min] was identified as the de-CA and de-Rha product of M1.

For M2, there is a Glu, not a Rha moiety, substituted at the C-6′ position by comparison with M1. Therefore, M2-15 or isomers [*m*/*z* 623.20 (C_29_H_35_O_15_), eluted at 23.14 and 26.08 min, respectively] were assigned as C-6′ de-Glu products of M2 and also confirmed as acteoside and isoacteoside, respectively. M2-4 [*m*/*z* 487.14587 (C_21_H_27_O_13_), eluted at 8.91 min] was identified as the de-Glu and de-HT product of M2. M2-3 [*m*/*z* 461.16669 (C_20_H_29_O_12_), eluted at 8.06 min] was identified as the de-CA and de-Glu products of M2 (corresponding to the fragment ion at *m*/*z* 461.16519). M2-2 [*m*/*z* 477.16132 (C_20_H_29_O_13_), eluted at 6.87 min], which is really close to the de-CA and de-Rha fragment ion of M2 (*m*/*z* 477.16013), is proposed to be the de-CA and de-Rha product of M2. The fragment ion at *m*/*z* 315.11 (C_14_H_19_O_8_, −162 Da) was formed by a loss of Glu (C_6_H_10_O_5_, −162 Da) and yielded product ions associated with Glu at *m*/*z* 179.06 (C_6_H_11_O_6_), 161.04 (C_6_H_9_O_5_), and 143.03 (C_6_H_7_O_4_). The diagnostic fragmentation from the HT moiety was also found at *m*/*z* 153.06 and 123.04.

Compared with M2, the structure of M3 has an additional acetyl group conjugated at the C-2′ position. Therefore, M3-18 [*m*/*z* 785.25085 (C_35_H_45_O_20_), eluted at 20.36 min] was assigned as the C-2′ de-Ac product of M3 and confirmed as the M2 isomer, which had identical fragments to M2 [*m*/*z* 785.25031 (C_35_H_45_O_20_), eluted at 16.73 min]. M3-2 [*m*/*z* 477.16122 (C_20_H_29_O_13_), eluted at 6.86 min] was assigned as C-2′ de-Ac, de-CA, and de-Rha products of M3 (corresponding to the fragment ion at *m*/*z* 477.16074). M3-1 [*m*/*z* 477.14020 (C_23_H_25_O_11_), eluted at 21.37 min] was assigned as the C-2′ de-Ac, de-Glu, and de-Rha product of M3 based on the HRAM of precursor and product ions. The fragment ion at *m*/*z* 315.11 (C_14_H_19_O_8_, -162 Da) was formed by a loss of CA (C_9_H_6_O_3_, −162 Da) and yielded product ions associated with CA at *m*/*z* 179.03, 161.02, and 135.04. M3-5 or isomers [*m*/*z* 623.20 (C_29_H_35_O_15_), eluted at 23.12 and 26.06 min, respectively] were assigned as the C-2′ de-Ac and de-Glu products of M2 and also confirmed as acteoside and isoacteoside, respectively. M3-6 [*m*/*z* 461.16653 (C_20_H_29_O_12_), eluted at 8.04 min] was assigned as the C-2′ de-Ac, de-CA, and de-Glu products of M3 (corresponding to the fragment ion at *m*/*z* 461.16684). M3-7 [*m*/*z* 315.10870 (C_14_H_19_O_8_), eluted at 8.07 min] was 146 Da (C_6_H_10_O_4_) less than M3-6, assigned as the de-Rha product of M3-6 (corresponding to the fragment ion at *m*/*z* 315.10870), and product ions associated with HT (*m*/*z* of 153.06 and 135.04) were formed by a loss of Glu (C_6_H_10_O_5_, -162 Da). M3-4 or isomers [*m*/*z* 623.22 (C_26_H_39_O_17_), eluted at 16.66 and 20.31 min, respectively] were assigned as the C-2′ de-Ac and de-CA products of M2 (corresponding to the fragment ion at *m*/*z* 623.22), and the major fragment ions of M3-4 or isomers (*m*/*z* of 477.16 and 461.17) were the same as M3 and the yielded product ions associated with Glu at *m*/*z* 179.06 (C_6_H_11_O_6_), 161.04 (C_6_H_9_O_5_), and 143.03 (C_6_H_7_O_4_).

Compared with M4, the structure of M3 had an additional Glc group conjugated at the C-6′ position. Therefore, M3-15 [*m*/*z* 665.20892 (C_31_H_37_O_16_), eluted at 30.01 min] was assigned as the de-Glu product of M3 and also confirmed as M4 [*m*/*z* 665.20874 (C_31_H_37_O_16_), eluted at 29.91 min]. M3-10 [*m*/*z* 503.17633 (C_22_H_31_O_13_), eluted at 12.33 min] was assigned as the de-CA product of M3-15 (corresponding to the fragment ion at *m*/*z* 503.17685), and the major fragment ions of M3-10 (*m*/*z* of 461.17 and 315.11) were also the same as M3 by a loss of CA. The diagnostic fragmentation from the HT moiety was found at *m*/*z* 153.06 and 123.04, suggesting that the fragment was the de-Glu and de-CA products of M3. M3-22 [*m*/*z* 519.15063 (C_25_H_27_O_12_), eluted at 31.05 min] was 146 Da (C_6_H_10_O_4_) less than M3-15 and also yielded product ions associated with CA at *m*/*z* 179.03, 161.02, and 135.04, suggesting that M3-22 was the de-Glu and de-Rha product of M3. M3-12 [*m*/*z* 529.15613 (C_23_H_29_O_14_), eluted at 11.13 min] was 136 Da (C_8_H_8_O_2_) less than M3-15 and also yielded product ions associated with CA at *m*/*z* 179.03, 161.02, and 135.04, suggesting that M3-12 was the de-Glu and de-HT product of M3.

Similarly, M4-5 [*m*/*z* 503.17694 (C_22_H_31_O_13_), eluted at 12.32 min] was assigned as the de-CA product of M4 (corresponding to the fragment ion at *m*/*z* 503.17511) and yielded the same fragment ions as from M4 by a loss of CA. M4-6 [*m*/*z* 461.16635 (C_20_H_29_O_12_), eluted at 8.03 min] was assigned as the de-CA and de-Ac product of M4 (corresponding to the fragment ion at *m*/*z* 461.16574). M4-22 or isomers [*m*/*z* 623.20 (C_29_H_35_O_15_), eluted at 23.06 and 26.11 min, respectively] were assigned as the de-Ac products of M4 and also confirmed as acteoside and isoacteoside, respectively. M4-8 [*m*/*z* 529.15521 (C_23_H_29_O_14_), eluted at 11.08 min] was 136 Da (C_8_H_8_O_2_) less than M4 and also yielded product ions associated with CA at *m*/*z* 179.03, 161.02, and 135.04, suggesting that M4-8 was the de-HT product of M4. M4-17 [*m*/*z* 519.15039 (C_25_H_27_O_12_), eluted at 31.11 min] was 146 Da (C_6_H_10_O_4_) less than M4, suggesting that it was the de-Rha product of M4. The fragment ions at *m*/*z* 477.14 (C_23_H_25_O_11_) and 357.12 (C_16_H_21_O_9_, −162 Da) were formed by the further loss of Ac (C_2_H_2_O, −42 Da) and CA (C_9_H_6_O_3_, −162 Da), and yielded product ions associated with CA at *m*/*z* 179.03, 161.02, and 135.04. Fragment ions at *m*/*z* 153.06 and 123.04 were attributed to the HT group. Therefore, M4-2 [*m*/*z* 357.11899 (C_16_H_21_O_9_), eluted at 9.36 min] was assigned as the de-CA product of M4-17 (corresponding to the fragment ion at *m*/*z* 357.11856) and M4-20 [*m*/*z* 477.13998 (C_23_H_25_O_11_), eluted at 21.24 min] was assigned as the de-Ac product of M4-17 (corresponding to the fragment ion at *m*/*z* 477.14072).

Finally, these four PhGs were all degraded to aglycone HT [*m*/*z* 153.06 (C_8_H_9_O_3_), eluted at 6.6 min] and CA [*m*/*z* 179.03 (C_9_H_7_O_4_), eluted at 12.3 min], and then CA underwent further metabolic process to form other microbial metabolites. The α′, β′-double bond of CA was easily reduced to the metabolite, 3,4-dihydroxybenzenepropionic acid [*m*/*z* 181.05 (C_9_H_9_O_4_), eluted at 10.3 min], and further dehydroxylated to 3-hydroxyphenylpropionic acid [*m*/*z* 165.06 (C_9_H_9_O_3_), eluted at 14.5 min]. These metabolite identifications were verified by comparison with authentic reference standards. Additionally, the acetylated product of CA [*m*/*z* 221.05 (C_11_H_9_O_5_), eluted at 15.8 min] showed the added mass of 42 Da over CA and the presence of a fragment ion at *m*/*z* 177.06 (C_10_H_9_O_3_) formed by a CO_2_ loss. Similarly, the acetylated product of 3,4-dihydroxybenzenepropionic acid also showed an added mass of 42 Da over 3,4-dihydroxybenzenepropionic acid at *m*/*z* 223.06 (C_11_H_11_O_5_) and the successive CO_2_ loss ion at *m*/*z* 179.07 (C_10_H_11_O_3_). In the same way, the sulfated product of 3,4-dihydroxybenzenepropionic acid [*m*/*z* 261.01 (C_9_H_9_O_7_S), 9.3 min] showed the added mass of 80 Da over 3,4-dihydroxybenzenepropionic acid and the presence of characteristic fragment ions at *m*/*z* 181.05 (C_9_H_9_O_4_) and 137.06 (C_8_H_9_O_2_) formed by losing a SO_3_ and CO_2_, respectively.

#### Phase I and II Metabolites

In this study, we proposed a strategy for rapidly characterizing the phase I and II metabolites of both parent compounds and their degradation products (see the section Degradation Products of Parent Compounds). The direct phase I and II metabolites of these compounds were relatively easy to characterize. First, with the benefit of HRAM data acquired by Orbitrap mass, these metabolites were compared by the mass and elemental composition differences of the precursors. Subsequently, the reasonable formula change could be determined based on the knowledge of transformations. Finally, the fragmentation pattern comparisons helped to confirm the characterizations and the distinctive fragmentation ions contributed to determining metabolites or isomers.

Moreover, most metabolites can go through degradation and further phase I and/or phase II metabolism. Such metabolites are always produced from multiple metabolic reactions from the parent compound. The proposed multiple crossover “fragmentation–degradation” comparisons between parent compound and their phase I and II metabolites help to rapidly characterize such metabolites (García-Reyes et al., 2007; Wang et al., 2009; López et al., 2016).

**Figure 5:**
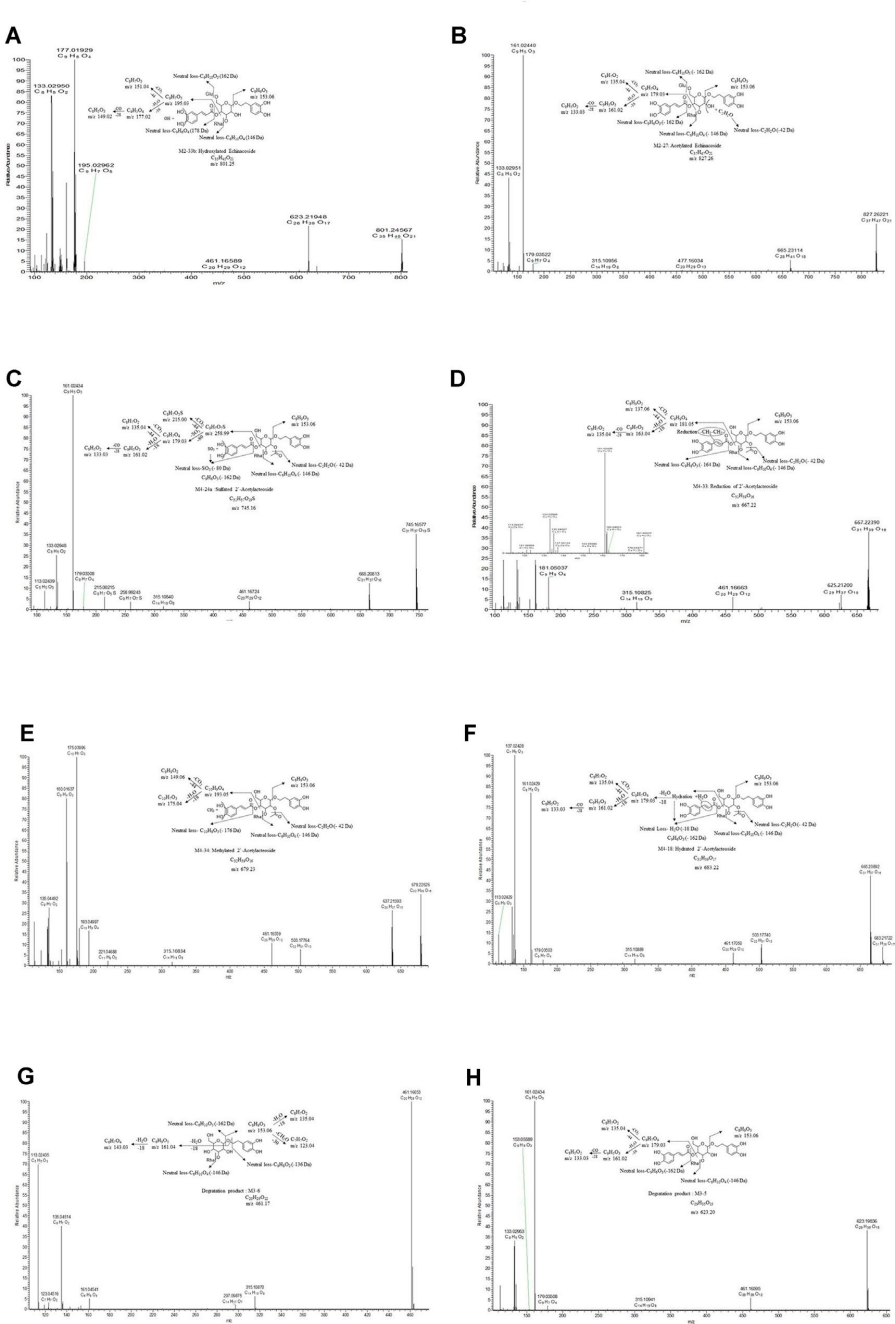
Proposed fragmentation pathways: **(A)** M2-33b (*m*/*z* 801); **(B)** M2-27 (*m*/*z* 827); **(C)** M4-24a (*m*/*z* 745); **(D)** M4-33 (*m*/*z* 667); **(E)** M4-34 (*m*/*z* 679); **(F)** M4-18 (*m*/*z* 683); **(G)** M3-6 (*m*/*z* 461); and **(H)** M3-5 (*m*/*z* 623).

The identification process of these metabolites is also automatically achieved by the FISh function in Compound Discoverer™ software. To better characterize the metabolites, those metabolites with the same metabolic pathway which had similar fragmentation patterns and diagnostic ions could be discussed together. Furthermore, some structural characterization of the metabolites can be achieved and described in detail as follows. The proposed chemical structures and fragmentation patterns of M2-33b, M2-27, M4-24a, M4-33, M4-34, M4-18, M3-6, and M3-5 are shown in **Figure 5**.

##### Hydroxylated Metabolite (+ O, 16 Da)

M1-7a and M1-7b (eluted at 17.26 and 21.82 min, respectively) with similar quasi-molecular ions at *m*/*z* 785.25 (C_35_H_45_O_20_) were 16 Da (+O) higher than M1, indicating that M1-7a and M1-7b were hydroxylated products of M1. Based on the subsequent fragmentation patterns, the hydroxylation site was partly confirmed. In the MS/MS spectra of M1-7a, the fragment ion at *m*/*z* 633.20 (C_27_H_37_O_17_) was formed by a loss of hydroxylated HT (C_8_H_8_O_3_, −152 Da). The presence of ions at *m*/*z* 169.05 (C_8_H_9_O_4_) and 151.04 (C_8_H_7_O_3_) corresponded to hydroxylated HT, as evidenced by the 16-Da (+O) difference from HT [*m*/*z* 153.06 (C_8_H_9_O_3_)], suggesting that the hydroxylation site of M1-7a was at the HT moiety. In the MS/MS spectra of M1-7b, the fragment ion at *m*/*z* 607.22 (C_26_H_39_O_16_) was formed by a loss of hydroxylated CA (C_9_H_6_O_4_, −178 Da). The presence of ions at *m*/*z* 195.03 (C_9_H_7_O_5_), 177.02 (C_9_H_5_O_4_), and 149.02 (C_8_H_5_O_3_) corresponded to hydroxylated CA, as evidenced from the 16-Da (+O) difference from CA [*m*/*z* 179.03 (C_9_H_7_O_4_)], suggesting that the hydroxylation site of M1-7b was at the CA moiety. Similarly, M2-33a and M2-33b [*m*/*z* 801.25 (C_35_H_45_O_21_), eluted at 13.06 and 15.39 min, respectively], M3-38 [*m*/*z* 843.25897 (C_37_H_47_O_22_), 16.54 min], and M4-21a and M4-21b [*m*/*z* 681.20 (C_31_H_37_O_17_), eluted at 20.30 and 24.14 min, respectively] could be deduced as the hydroxylated HT or CA product of M2, M3, and M4, respectively.

Compared with the defined degradation products, M1-23a and M1-23b [*m*/*z* 639.19 (C_29_H_35_O_16_), eluted at 16.12 and 20.27 min, respectively] were 16 Da (+O) higher than the defined de-Rha degradation product of M1 [*m*/*z* 623.20 (C_29_H_35_O_15_)], suggesting that M1-23a and M1-23b were subsequent hydroxylated products after de-Rha degradation of M1. When matching the fragments of M1-7a and M1-7b, a similar fragmentation pattern supported such a characterization and diagnostic ions corresponding to the hydroxylated HT (*m*/*z* of 169.05 and 151.04) or CA moiety (*m*/*z* of 195.03, 177.02, and 149.02) were also demonstrated in M1-23a and M1-23b, respectively. Similarly, M2-17a and 17b [*m*/*z* 639.19 (C_29_H_35_O_16_), eluted at 16.08 and 20.31 min], M3-28a and 28b [*m*/*z* 801.25 (C_35_H_45_O_21_), eluted at 13.02 and 15.46 min, respectively], M3-17 [*m*/*z* 681.20 (C_31_H_37_O_17_), eluted at 20.26 min], M3-20a and 20b [*m*/*z* 639.19 (C_29_H_35_O_16_), eluted at 16.11 and 20.22 min], M3-42 [*m*/*z* 477.16132 (C_20_H_29_O_13_), eluted at 5.21 min], M4-29a and 29b [*m*/*z* 639.19 (C_29_H_35_O_16_), eluted at 16.11 and 20.24 min], and M4-4 [*m*/*z* 519.17 (C_22_H_31_O_14_), eluted at 7.76 min] also deduced the subsequent hydroxylated HT or CA product corresponding to the detected degradation products of M2, M3, and M4, respectively.

##### Hydrated Metabolites (+ H_2_O, +18 Da)

M1-9 [*m*/*z* 787.26459 (C_35_H_47_O_20_), eluted at 16.76 min] was 18 Da (+H_2_O) higher than M1. In the MS/MS spectrum of M1-9, the fragment ion at *m*/*z* 769.26 was formed by a loss of H_2_O from the quasi-molecular ion. Additionally, the MS/MS spectrum showed a series of fragment ions at *m*/*z* 607.22, 461.17, 179.03, and 161.02, which were the same as the characteristic fragment ions of M1. Thus, M1-9 was deduced as the hydrated metabolite of M1, and the hydration site was also at the α′, β′-d-double bond of the CA moiety with the presence of ion at *m*/*z* 137.02 (C_7_H_5_O_3_). Similarly, M3-39 [*m*/*z* 845.27061 (C_37_H_49_O_22_), eluted 17.89 min] and M4-18 [*m*/*z* 683.21722 (C_31_H_39_O_17_), eluted 22.49 min] were deduced as the hydrated metabolite of M3 and M4, respectively, and the hydration site was also at the α′, β′-d-double bond.

M3-16 [*m*/*z* 683.22046 (C_31_H_39_O_17_), eluted at 22.49 min] was 18 Da (+H_2_O) higher than the defined de-Glu degradation product of M3 [*m*/*z* 665.21 (C_31_H_37_O_16_)] and further formed the same fragment ions by a loss of H_2_O, suggesting that M3-16 was the subsequent hydrated product after de-Glu degradation of M3. When matching the fragments of M3-39, a similar fragmentation pattern supported such a characterization and the hydration site was also at the α′, β′-d-double bond of the CA moiety with the presence of ion at *m*/*z* 137.02 (C_7_H_5_O_3_). Similarly, M3-40 [*m*/*z* 803.26263 (C_35_H_47_O_21_), eluted at 14.17 min] and M3-33 [*m*/*z* 641.20837 (C_29_H_37_O_16_), eluted at 15.54 min] were deduced subsequent to the hydrated CA product corresponding to the detected degradation products of M3, respectively.

##### Hydroxylated and Hydrated Metabolites (+ O and + H_2_O, 34 Da)

M1-6 [*m*/*z* 803.26215 (C_35_H_47_O_21_), eluted at 14.15 min] was 18 Da (+H_2_O) higher than M1-7a or M1-7b in molecular weight, indicating that M1-6 was the hydrated product of M1-7a or M1-7b. In the MS/MS spectrum of M1-6, the fragment ion at *m*/*z* 785.25 was formed by a loss of H_2_O from the quasi-molecular ion. Additionally, the MS/MS spectrum showed a series of fragment ions at *m*/*z* 607.22, 195.03, 177.02, 149.02, and 153.06, which were the same as the characteristic fragment ions of M1-7b (hydroxylated CA product of M1). Thus, M1-6 was deduced as the hydrated and hydroxylated CA product of M1 with the presence of ion at *m*/*z* 137.02 (C_7_H_5_O_3_).

M4-15 [*m*/*z* 699.21368 (C_31_H_39_O_18_), eluted at 16.42 min] was 16 Da (+O) higher than M4-18, indicating that M4-15 was the hydroxylated product of M4-18 (hydrated CA product of M4). In the MS/MS spectra of M4-15, the fragment ion at *m*/*z* 681.20 (C_31_H_37_O_17_) was formed by a loss of H_2_O from the quasi-molecular ion and showed similar product ion at *m*/*z* 503.18 (C_31_H_37_O_17_) by further loss of hydroxylated CA (C_9_H_6_O_4_, −178 Da) and yielded product ions associated with hydroxylated CA (*m*/*z* of 195.03, 177.02, or 149.02); thus, M4-15 could be deduced as the hydrated and hydroxylated CA product of M4.

M3-24 [*m*/*z* 657.20441 (C_29_H_37_O_17_), eluted at 12.51 min] was 18 Da (+H_2_O) higher than M3-20a or 20b [*m*/*z* 639.19 (C_29_H_35_O_16_)] in molecular weight and further formed the same fragment ions of M3-20b by a loss of H_2_O, indicating that M3-24 was the hydrated CA product of M3-20b (a subsequent hydroxylated CA product after de-Ac and de-Glu degradation of M3) with the presence of ion at *m*/*z* 137.02 (C_7_H_5_O_3_).

##### Hydroxylated and Dehydrogenated Metabolites (+ O and − H_2_, +14 Da)

M1-17 [*m*/*z* 783.23444 (C_35_H_43_O_20_), eluted at 19.15 min] was 2 Da (−H_2_) lower than M1-7a or M1-7b in molecular weight, indicating that M1-17 was the dehydrogenated product of M1-7a or M1-7b. In the MS/MS spectrum of M1-17, the fragment ion at *m*/*z* 633.20 (C_27_H_37_O_17_) was formed by a loss of dehydrogenated and hydroxylated HT (C_8_H_6_O_3_, −150 Da). The presence of ions at *m*/*z* 167.03 (C_8_H_7_O_4_) and 149.02 (C_8_H_5_O_3_) corresponding to dehydrogenated and hydroxylated HT, as evidenced by the 2-Da (−H_2_) difference from hydroxylated HT (*m*/*z* 169.05, C_8_H_9_O_4_), suggested that the dehydrogenation site of M1-7a was at the hydroxylated HT moiety of M1-7a. Similarly, M2-35 [*m*/*z* 799.23041 (C_35_H_43_O_21_), eluted at 14.95 min], M3-26 [*m*/*z* 841.23883 (C_37_H_45_O_22_), eluted at 18.45 min], and M4-27 [*m*/*z* 679.18811 (C_31_H_35_O_17_), eluted at 23.24 min] could be deduced as the hydroxylated and dehydrogenated HT products of M2, M3, and M4, respectively.

M1-24 [*m*/*z* 637.17773 (C_29_H_33_O_16_), eluted at 18.93 min] was 2 Da (−H_2_) lower than M1-23a or M1-23b [*m*/*z* 639.19 (C_29_H_35_O_16_)], indicating that M1-24 was the dehydrogenated product of M1-23a or M1-23b.When matching the fragments of M1-17, a similar fragmentation pattern supported such a characterization and diagnostic ions corresponding to dehydrogenated and hydroxylated HT (*m*/*z* of 167.03 and 149.02) were also demonstrated in M1-24, suggesting that M1-17 was subsequently dehydrogenated and hydroxylated products after de-Rha degradation of M1 [*m*/*z* 623.20 (C_29_H_35_O_15_)]. M1-19 [*m*/*z* 475.14523 (C_20_H_27_O_13_), eluted at 7.04 min] was assigned as a subsequently dehydrogenated and hydroxylated product after de-Rha and de-CA degradation of M1 [*m*/*z* 461.17 (C_20_H_29_O_12_)]. The matched fragment ion of M1-17 [*m*/*z* 475.14514 (C_20_H_27_O_13_)] by further loss of Rha and CA and diagnostic ions corresponding to dehydrogenated and hydroxylated HT (*m*/*z* of 167.03 or 149.02) were also demonstrated in M1-19. Similarly, M2-18 [*m*/*z* 637.17871 (C_29_H_33_O_16_), eluted at 19.03 min], M3-21 [*m*/*z* 637.17761 (C_29_H_33_O_16_), eluted at 19.03 min], M3-29 [*m*/*z* 799.23041 (C_35_H_43_O_21_), eluted at 14.92 min], M3-27 [*m*/*z* 679.18854 (C_31_H_35_O_17_), eluted at 23.27 min], M4-36 [*m*/*z* 637.17780 (C_29_H_33_O_16_), eluted at 18.99 min], and M4-13 [*m*/*z* 517.15552 (C_22_H_29_O_14_), eluted at 10.13 min] were the subsequently deduced dehydrogenated and hydroxylated HT products corresponding to the detected degradation products of M1, M2, M3, and M4, respectively.

##### Dihydroxylation (+ 2O, +32 Da)

M1-16 [*m*/*z* 801.24530 (C_35_H_45_O_21_), eluted at 17.84 min] was 32 Da (+2O) higher than M1, which indicated that M1-16 was the dihydroxylated product of M1. In the MS/MS spectra of M1-16, the fragment ion at *m*/*z* 607.22 (C_26_H_39_O_16_) was a dihydroxylated CA moiety loss (C_9_H_6_O_5_, −194 Da) from the quasi-molecular ion, which suggested that the dihydroxylation site was at the CA moiety. In addition, the fragment ion at *m*/*z* 193.01 (C_9_H_5_O_5_) was formed by a loss of H_2_O from dihydroxylated CA (C_9_H_7_O_6_), and product ions at *m*/*z* 165.02 (C_8_H_5_O_4_) and 149.02 (C_8_H_5_O_3_) were formed by further loss of CO and CO_2_. Similarly, M2-39 [*m*/*z* 817.24133 (C_35_H_45_O_22_), eluted at 13.81 min] and M4-32 [*m*/*z* 697.19904 (C_31_H_37_O_18_), eluted at 19.07 min] could be deduced as the dihydroxylated CA products of M2 and M4, respectively.

M2-40 [*m*/*z* 655.18976 (C_29_H_35_O_17_), eluted at 18.23 min] was 32 Da (+2O) higher than the defined de-Glu degradation product of M2 [*m*/*z* 623.20 (C_29_H_35_O_15_)] and formed the same fragment ions (*m*/*z* of 461.17 and 315.11) by further loss of dihydroxylated CA and Rha, suggesting that M2-40 was the subsequent dihydroxylated product after the de-Glu degradation product of M2. When matching the fragments of M2-39, the similar fragmentation pattern supported such a characterization, and diagnostic ions corresponding to the dihydroxylated CA moiety (*m*/*z* of 193.01, 165.02, and 149.02) were also demonstrated in M2-40. Similarly, M3-41 [m/z 817.23987 (C_35_H_45_O_22_), eluted at 13.73 min] and M3-32 [*m*/*z* 655.18762 (C_29_H_35_O_17_), eluted at 15.21 min] could be deduced as the dihydroxylated CA products corresponding to the detected degradation products of M3, respectively.

##### Methylated Metabolites (+ CH_2_, +14 Da)

M2-36 [*m*/*z* 799.26758 (C_36_H_47_O_20_), eluted at 23.67 min] was 14 Da (+CH_2_) higher than M2 in molecular weight, indicating that M2-36 was the methylated product of M2. In the MS/MS spectrum of M2-36, the fragment ion at *m*/*z* 623.22 (C_26_H_39_O_17_) was formed by a loss of methylated CA (C_10_H_8_O_3_, −176 Da) from the quasi-molecular ion. In addition, the presence of fragment ions at *m*/*z* 193.05 (C_10_H_9_O_4_) corresponding to methylated CA, as evidenced by the 14-Da (+CH_2_) difference from CA (*m*/*z* 179.03, C_9_H_7_O_4_) and product ions at *m*/*z* 175.04 (C_10_H_7_O_3_) and 149.06 (C_9_H_9_O_2_) formed by further loss of H_2_O and CO_2_, suggested that the methyl site was at one of the hydroxyls of the CA moiety. Similarly, M4-34 [*m*/*z* 679.22626 (C_32_H_39_O_16_), eluted at 33.02 min] was deduced as the methylated CA product of M4.

M1-25 [*m*/*z* 637.21503 (C_30_H_37_O_15_), 28.18 min] was 14 Da (+CH_2_) higher than the defined de-Rha degradation product of M1 [*m*/*z* 623.20 (C_29_H_35_O_15_)] and formed the same fragment ion at *m*/*z* 461.17 (C_20_H_29_O_12_) by a loss of methylated CA (C_10_H_8_O_3_, −176 Da), and diagnostic ions corresponding to methylated CA (*m*/*z* of 193.05, 175.04, and 149.06) were also demonstrated in M1-25, suggesting that M1-25 was the subsequent methylated CA product after de-Rha degradation of M1. Similarly, M2-16 [*m*/*z* 637.21271 (C_30_H_37_O_15_), eluted at 28.20 min] and M4-35 [*m*/*z* 637.21411 (C_30_H_37_O_15_), eluted at 28.25 min] were deduced as the methylated CA products corresponding to the detected degradation products of M2 and M4, respectively.

##### Hydrated and Methylated Metabolites (+ H_2_O and + CH_2_, +32 Da)

M1-10 [*m*/*z* 801.28125 (C_36_H_49_O_20_), eluted at 24.24 min] was 14 Da (+CH_2_) higher than M1-9 in molecular weight, indicating that M1-10 was the methylated product of M1-9. In the MS/MS spectrum of M1-10, the fragment ion at *m*/*z* 769.26 (C_35_H_45_O_19_) was formed by further loss of CH_2_ and H_2_O from the quasi-molecular ion and other fragment ions were demonstrated (*m*/*z* of 607.22, 461.17 and 153.06), which was consistent with the characteristic fragment ions of M1-9.

M2-37 [*m*/*z* 817.27893 (C_36_H_49_O_21_), eluted at 17.59 min] was 18 Da (+H_2_O) higher than M2-36 in molecular weight, indicating that M2-37 was the hydrated product of M2-36. In the MS/MS spectrum of M2-37, the fragment ion at *m*/*z* 785.25 (C_35_H_45_O_20_) was formed by the further loss of CH_2_ and H_2_O from the quasi-molecular ion. In addition, the presence of fragment ions corresponding to methylated CA (*m*/*z* of 193.05 and 149.06) was also demonstrated in M2-37.

M2-38 [*m*/*z* 655.22577 (C_30_H_39_O_16_), eluted at 21.65 min] was 18 Da (+H_2_O) higher than M2-16 and formed the same fragment ions by further loss of H_2_O and methylated CA (C_10_H_8_O_3_, −176 Da). The fragment ion at *m*/*z* 623.20 (C_29_H_35_O_15_), corresponding to the de-Glu degradation product of M2 [*m*/*z* 623.20 (C_29_H_35_O_15_)], was formed by the further loss of CH_2_ and H_2_O. When matching the fragments of M2-37, a similar fragmentation pattern also supported such a characterization, and diagnostic ions corresponding to methylated CA (*m*/*z* of 193.05 and 149.06) were also demonstrated in M2-38. Similarly, M3-31 [*m*/*z* 817.27802 (C_36_H_49_O_21_), eluted at 17.58 min] and M3-34 [*m*/*z* 655.22479 (C_30_H_39_O_16_), eluted at 21.67 min] were deduced as the hydrated and methylated CA products corresponding to the detected degradation product of M3, respectively.

##### Sulfated Metabolites (+ SO_3_, +80 Da)

M1-11a (eluted at 22.77 min), M1-11b (eluted at 12.27 min), and M1-11c (eluted at 23.53 min) exhibited the same molecular formula of C_35_H_45_O_22_S (*m*/*z* 849.21) and were 80 Da (+SO_3_) higher than M1. M1-11a, M1-11b, and M1-11c were determined to be the sulfated products of M1. In the MS/MS spectra, M1-11a, M1-11b, and M1-11c showed different fragment ions, indicating that M1-11a, M1-11b, and M1-11c were sulfated at different sites of the structure. In the MS/MS spectra of M1-11c, the fragment ion at *m*/*z* 769.26 was formed by a loss of SO_3_ from the quasi-molecular ion. Additionally, other fragment ions from M1-11c were found (*m*/*z* of 607.22, 461.17, 179.03, and 161.02), which were consistent with the characteristic fragment ions of M1, indicating that the sulfation site was at the C-2′ position. In the MS/MS spectra of M1-11a, the fragment ion at *m*/*z* 215.00 (C_8_H_7_O_5_S) was presumed to be a CO_2_ loss from the sulfated CA [*m*/*z* 258.99 (C_9_H_7_O_7_S)] as evidenced by the 80-Da (+SO_3_) difference from CA [*m*/*z* 179.03 (C_9_H_7_O_4_)] and the presence of characteristic fragment ions of CA (*m*/*z* 179.03, 161.02, and 135.04) formed by a loss of SO_3_, indicating that the sulfation site of M1-11a was at one of the hydroxyls of the CA moiety. In the MS/MS spectra of M1-11b, the fragment ion at *m*/*z* 687.18 (C_26_H_39_O_19_S) was a CA moiety loss from the quasi-molecular ion, which suggested that the sulfate site was not at the CA moiety. In addition, the fragment ion at *m*/*z* 215.00 (C_8_H_7_O_5_S) was presumed to be a H_2_O loss from sulfated HT [*m*/*z* 233.01 (C_8_H_9_O_6_S)], as evidenced by the 80-Da (+SO_3_) difference from HT [*m*/*z* 153.06 (C_8_H_9_O_3_)], and confirming that the sulfation site of M1-11b was at one of the hydroxyls of the HT moiety. Similarly, M4-24a (22.44 min), M4-24b (27.76 min), and M4-24c (16.69 min), with a similar quasi-molecular ion at *m*/*z* 745.16 (C_31_H_37_O_19_S), were deduced as the sulfated products of M4, and the sulfation sites of M4-24b, 24a, and 24c were at the C-2′ position, one of the hydroxyls of the CA moiety, and one of the hydroxyls of the HT moiety, respectively.

M3-13a, M3-13b, and M3-13c [*m*/*z* 865.21 (C_35_H_45_O_23_S), eluted at 15.53, 16.00, and 12.93 min, respectively] were 80 Da (+SO_3_) higher than the defined de-Ac degradation product of M3 [*m*/*z* 785.25085 (C_35_H_45_O_20_)], suggesting that they were subsequent sulfated products after de-Ac degradation of M3, and the sulfation sites of M3-13a, M3-13b, and M3-13c were deduced as a sulfated HT, sulfated C-2′ position, or sulfated CA product after de-Ac degradation of M3 by comparison with the fragments, respectively. Similarly, M3-19a, M3-19b, and M3-19c [*m*/*z* 703.15 (C_29_H_35_O_18_S), eluted at 15.59, 19.21, and 21.39 min, respectively], and M4-3 [*m*/*z* 583.13373 (C_22_H_31_O_16_S), eluted at 11.25 min] and M4-19 [*m*/*z* 703.15723 (C_29_H_35_O_18_S), eluted at 21.35 min] were deduced as a sulfated HT, sulfated C-2′ position, or sulfated CA product corresponding to the detected degradation products of M3 and M4, respectively.

##### Reduced Metabolites (+ 2H, +2 Da)

M1-12 [*m*/*z* 771.27032 (C_35_H_47_O_19_), eluted at 23.70 min] was 2 Da (+ 2H) higher than M1, which indicated that M1-12 was the reduced product of M1. In the MS/MS spectrum of M1-12, the fragment ion at *m*/*z* 607.22 (C_26_H_39_O_16_) was formed through the loss of reduced CA (C_9_H_8_O_3_, −164 Da). Additionally, the presence of fragment ions at *m*/*z* 137.06 (C_8_H_9_O_2_) was presumed to be a CO_2_ loss from reduced CA [*m*/*z* 181.05 (C_9_H_9_O_4_)], as evidenced by the 2-Da (+2H) difference from CA [*m*/*z* 179.03 (C_9_H_7_O_4_)], implying that the reduction occurred on the α′,β′-double bond of the CA moiety. Similarly, M2-34 [*m*/*z* 787.26796 (C_35_H_47_O_20_), eluted at 16.25 min], M3-37 [*m*/*z* 829.27435 (C_37_H_49_O_21_), eluted at 24.68 min], and M4-33 [*m*/*z* 667.21930 (C_31_H_37_O_17_), eluted at 28.72 min] were deduced as the reduced CA products of M2, M3, and M4, respectively.

M3-30 [*m*/*z* 667.22681 (C_31_H_39_O_16_), eluted at 28.76 min] was 2 Da (+2H) higher than the defined de-Glu degradation product of M3 [*m*/*z* 665.20892 (C_31_H_37_O_16_)] and formed the same fragment ion at *m*/*z* 461.17 (C_20_H_29_O_12_) by further loss of Ac (C_2_H_2_O) and reduced CA (C_9_H_8_O_3_), suggesting that M3-30 was the reduced CA product after de-Glu degradation of M3.

##### Dehydroxylated Metabolites (− O, −16 Da)

M2-26a and M2-26b (eluted at 19.71 and 25.01 min, respectively) have a similar quasi-molecular ion at *m*/*z* 769.25 (C_35_H_45_O_19_) that was 16 Da (−O) lower than M2 in molecular weight, indicating that M2-26a and M2-26b were the dehydroxylated products of M2. In the MS/MS spectrum of M2-26a, the fragment ion at *m*/*z* 623.22 (C_26_H_39_O_17_) was formed by a loss of dehydroxylated CA (C_9_H_6_O_2_, −146 Da). In addition, the fragment ions at *m*/*z* 163.04 (C_9_H_7_O_3_), 145.03 (C_9_H_5_O_2_), and 119.05 (C_8_H_7_O) corresponded to dehydroxylated CA, as evidenced by the 16 Da (−O) difference from CA [*m*/*z* 179.03 (C_9_H_7_O_4_)], suggesting that the dehydroxylation site of M2-26a was at the CA moiety. In the MS/MS spectra of M2-26b, the fragment ion at *m*/*z* 607.22 (C_26_H_39_O_16_) was formed by a loss of CA (C_9_H_6_O_3_, −162 Da) and yielded product ions associated with CA at *m*/*z* 179.03, 161.02, and 135.04. The presence of ions at *m*/*z* 137.06 (C_8_H_9_O_2_) and 119.05 (C_8_H_7_O) associated with dehydroxylated HT, as evidenced by the 16 Da (−O) difference from HT [*m*/*z* 153.06 (C_8_H_9_O_3_)], suggesting that the dehydroxylation site of M2-26b was at the HT moiety. Similarly, M4-25 [*m*/*z* 649.21411 (C_31_H_37_O_15_), eluted at 33.57 min] was deduced as the dehydroxylated CA product of M4.

M2-14a and M2-14b [*m*/*z* 607.20 (C_29_H_35_O_14_), eluted at 26.62 and 29.22 min, respectively] were 16 Da (−O) lower than the defined de-Glu degradation of M2 [*m*/*z* 623.20 (C_29_H_35_O_15_)] and assigned as subsequent dehydroxylated products after de-Glu degradation of M2. When matching the fragments of M2-26a and M2-26b, a similar fragmentation pattern supported such a characterization, and diagnostic ions corresponding to dehydroxylated CA (*m*/*z* of 163.04, 145.03, and 119.05) or the dehydroxylated HT moiety (*m*/*z* of 137.06 and 119.05) were also found in M2-14a and M2-14b, respectively. Similarly, M3-23 [*m*/*z* 649.21 (C_31_H_37_O_15_), eluted at 32.62 min], M3-25a [*m*/*z* 607.20 (C_29_H_35_O_14_), eluted at 26.58 min], and M3-25b [*m*/*z* 607.20 (C_29_H_35_O_14_), eluted at 29.20 min], and M4-7 [*m*/*z* 461.14 (C_23_H_25_O_10_), eluted at 25.13 min], M4-11 [*m*/*z* 445.17 (C_20_H_29_O_11_), eluted at 12.24 min], M4-23a [*m*/*z* 607.20 (C_29_H_35_O_14_), eluted at 26.56 min], and M4-23b [*m*/*z* 607.20 (C_29_H_35_O_14_), eluted at 29.17 min] were also deduced as the dehydroxylated CA or HT products corresponding to the detected degradation products of M3 and M4, respectively.

##### Acetylated Metabolites (+ C_2_H_2_O, +42 Da)

M1-13a (eluted at 30.41 min), M1-13b (eluted at 24.73 min), and M1-13c (eluted at 29.52 min) exhibited the same molecular formula of C_37_H_47_O_20_ (*m*/*z* 811.26) and was 42 Da (+C_2_H_2_O) higher than M1. M1-13a, M1-13b, and M1-13c were determined to be the acetylated products of M1. In the MS/MS spectra, M1-13a, M1-13b, and M1-13c showed different fragment ions, indicating that M1-13a, M1-13b, and M1-13c were acetylated at different sites of the structure. In the MS/MS spectra of M1-13a, the fragment ion at *m*/*z* 769.26 was formed by a loss of C_2_H_2_O from the quasi-molecular ion. Additionally, other fragment ions from M1-13a were demonstrated (*m*/*z* of 607.22, 179.03, 161.02, and 153.06), which were consistent with the characteristic fragment ions of M1, thus indicating that the acetylation site was at the C-2′ position. In the MS/MS spectra of M1-13c, the fragment ions at *m*/*z* 203.03 (C_11_H_7_O_4_) and 177.06 (C_10_H_9_O_3_) were presumed to be due to a loss of H_2_O and CO_2_ from the acetylated CA (C_11_H_9_O_5_) and other characteristic fragment ions of CA (*m*/*z* 179.03, 161.02, and 135.04) formed by a loss of C_2_H_2_O, indicating that the acetylation site of M1-13c was at one of the hydroxyls of the CA moiety. In the MS/MS spectra of M1-13b, the presence of fragment ion at *m*/*z* 195.07 (C_10_H_9_O_4_) and other characteristic fragment ions of CA (*m*/*z* 179.03, 161.02, and 135.04) also suggested that the acetylation site of M1-13b was at one of the hydroxyls of the HT moiety. Similarly, M2-27 or isomers [*m*/*z* 827.26 (C_37_H_47_O_21_), eluted at 23.64 and 26.27 min, respectively] and M4-26 [*m*/*z* 707.21887 (C_33_H_39_O_17_), eluted at 34.32 min] could be deduced as the acetylated C-2′ position products of M2 and M4, respectively.

M1-18 [*m*/*z* 665.20905 (C_31_H_37_O_16_), eluted at 29.90 min] was 42 Da (+C_2_H_2_O) higher than the defined de-Rha degradation of M1 [*m*/*z* 623.20 (C_29_H_35_O_15_)] and formed the same fragment ions by further loss of Ac and CA. The similar fragmentation pattern of M1-13a also suggested that M1-13a was acetylated at the C-2′ position of the product after de-Rha degradation of M1. Similarly, M2-10 [*m*/*z* 503.18 (C_22_H_31_O_13_), eluted at 12.43 min], M2-11 [*m*/*z* 519.17 (C_22_H_31_O_14_), eluted at 10.88 min], M2-12 [*m*/*z* 529.16 (C_23_H_29_O_14_), eluted at 11.09 min], M2-13 [*m*/*z* 519.15 (C_25_H_27_O_12_), eluted at 31.17 min], M2-19 [*m*/*z* 665.23 (C_28_H_41_O_18_), eluted at 23.61 min], M2-20 [*m*/*z* 665.21 (C_31_H_37_O_16_), eluted at 31.82 min], M4-10 [*m*/*z* 571.17 (C_25_H_31_O_15_), eluted at 12.18 min], and M4-16 [*m*/*z* 545.19 (C_24_H_33_O_14_), eluted at 15.41 min] were also deduced as the acetylated C-2′ position product corresponding to the detected degradation products of M2 and M4, respectively.

##### Hydroxylated, Dehydrogenated, and Acetylated Metabolites (+ O and - H_2_ and +C_2_H_2_O, + 56 Da)

M2-42 [*m*/*z* 841.24060 (C_37_H_45_O_22_), eluted at 18.53 min] was 42 Da (+C_2_H_2_O) higher than M2-35, which indicated that M2-42 was the acetylated product of M2-35. In the MS/MS spectrum of M2-42, the fragment ions at *m*/*z* 679.21 (C_28_H_39_O_19_) were formed by the further loss of CA (C_9_H_6_O_3_, −162 Da). Additionally, the presence of fragment ions at *m*/*z* 179.03 (C_9_H_7_O_4_), 167.03 (C_8_H_7_O_4_), and 161.02 (C_9_H_5_O_3_) was consistent with the characteristic fragment ions of M2-35, indicating that the dehydrogenation site was at the hydroxylated HT moiety and the acetylation site was at the C-2′ position.

M2-22 [*m*/*z* 679.18506 (C_31_H_35_O_17_), eluted at 23.31 min] was 42 Da (+C_2_H_2_O) higher than M2-18 [*m*/*z* 637.17871 (C_29_H_33_O_16_)]. The similar fragmentation pattern and diagnostic ions corresponding to dehydrogenated and hydroxylated HT (*m*/*z* of 167.03 and 149.02) were also detected in M2-22, suggesting that it was the acetylated product of M2-18.

##### Reduced and Acetylated Metabolites (+ H_2_ and + C_2_H_2_O, +44 Da)

M2-41 [*m*/*z* 829.27435 (C_37_H_49_O_21_), eluted at 24.68 min] was 42 Da (+C_2_H_2_O) higher than M2-34, which indicated that M2-41 was the acetylated product of M2-34. In the MS/MS spectrum of M2-41, the fragment ions at *m*/*z* 665.23 (C_28_H_41_O_18_) and 623.22 (C_26_H_39_O_17_) were formed by the further loss of reduced CA (C_9_H_8_O_3_, −164 Da) and Ac (C_2_H_2_O, −42 Da). Additionally, the presence of fragment ions at *m*/*z* 181.05 (C_9_H_9_O_4_) and 137.06 (C_8_H_9_O_2_) indicated that the reduction occurred on the α′, β′-double bond of the CA moiety and the acetylation site was at the C-2′ position.

M2-21 [*m*/*z* 667.21985 (C_31_H_39_O_16_), eluted at 28.74 min] was 2 Da (+H_2_) higher than M2-20 [*m*/*z* 665.20886 (C_31_H_37_O_16_), eluted at 31.82 min] and formed fragment ions at *m*/*z* 625.21 (C_29_H_37_O_15_) and 503.17 (C_22_H3_1_O_13_) by further loss of Ac (C_2_H_2_O) and reduced CA (C_9_H_8_O_3_). The similar fragmentation pattern of M2-41 and diagnostic ions corresponding to reduced CA (*m*/*z* of 181.05, 163.04, and 137.06) were also demonstrated in M2-21, suggesting that M2-41 was the reduced CA product of M2-20.

##### Hydrated and Acetylated Metabolites (+ H_2_O and + C_2_H_2_O, +60 Da)

M2-28 [*m*/*z* 845.27271 (C_37_H_49_O_22_), eluted at 17.92 min] was 18 Da (+H_2_O) higher than M2-27 or isomers, indicating that M2-28 was the hydrated product of M2-27 or isomers. In the MS/MS spectrum of M2-28, the fragment ion at *m*/*z* 827.26 was formed by a loss of H_2_O from the quasi-molecular ion. Additionally, the MS/MS spectrum showed a series of fragment ions at *m*/*z* 665.23, 623.22, 477.16, 315.11, 179.03, and 153.06, which were the same as the characteristic fragment ions of M2-27 or isomers. Thus, M2-28 could be deduced as the hydrated CA product of M2-27 or isomers with the presence of ions at *m*/*z* 137.02 (C_7_H_5_O_3_).

M2-24 or isomers [*m*/*z* 683.22 (C_31_H_39_O_17_), eluted at 20.77 and 22.55 min] were 18 Da (+H_2_O) higher than M2-20 [*m*/*z* 665.21 (C_31_H_37_O_16_)], corresponding to the fragment ion at *m*/*z* 665.21 (C_31_H_37_O_16_) by a loss of H_2_O, indicating that M2-24 or isomers were the hydrated products of M2-20. When matching the fragments of M2-28, the similar fragmentation pattern supported such a characterization and the hydration site was also at the α′, β′-d-double bond of the CA moiety with the presence of ion at *m*/*z* 137.02 (C_7_H_5_O_3_).

##### Sulfated and Acetylated Metabolites (+ SO_3_ and + C_2_H_2_O, +122 Da)

M2-29a and M2-29b [*m*/*z* 907.22 (C_37_H_47_O_24_S), eluted at 24.41 and 20.84 min, respectively] were 80 Da (+SO_3_) higher than M2-27 or isomers, indicating that M2-29a and M2-29b were the sulfated products of M2-27 or isomers. In the MS/MS spectra of M2-29a, the fragment ion at *m*/*z* 215.00 (C_8_H_7_O_5_S) presumed to be a CO_2_ loss from the sulfated CA [*m*/*z* 258.99 (C_9_H_7_O_7_S)], indicating that M2-29a was one of the sulfated products of M2-27 and the sulfation site was at the CA moiety. In the MS/MS spectra of M2-29b, the fragment ion at *m*/*z* 215.00 (C_8_H_7_O_5_S) was presumed to be a H_2_O loss from sulfated HT [*m*/*z* 233.01 (C_8_H_9_O_6_S)] and confirmed that the sulfation site was at the HT moiety.

##### Hydroxylated and Acetylated Metabolites (+ O and + C_2_H_2_O, +58 Da)

M2-30 [*m*/*z* 843.25507 (C_37_H_47_O_22_), eluted at 16.39 min] was 16 Da (+O) higher than M2-27 or isomers, indicating that M2-30 was the hydroxylated product of M2-27 or isomers. In the MS/MS spectra of M2-30, the fragment ion at *m*/*z* 665.23 (C_27_H_37_O_17_) was formed through the loss of hydroxylated CA (C_9_H_6_O_4_, −178 Da). The presence of ions at *m*/*z* 195.03 (C_9_H_7_O_5_) and 177.02 (C_9_H_5_O_4_) also suggested that the hydroxylation site of M2-30 was at the CA moiety.

M2-23a and M2-23b (eluted at 20.35 and 24.21 min, respectively) had a similar quasi-molecular ion at *m*/*z* 681.20 (C_31_H_37_O_17_) and were 16 Da (+O) higher than M2-20 [*m*/*z* 665.21 (C_31_H37O_16_)]. M2-23a was also 42 Da (+C_2_H_2_O) higher than M2-17a [*m*/*z* 639.19 (C_29_H_35_O_16_)]; the similar fragmentation pattern supported such a characterization, and diagnostic ions corresponding to the hydroxylated HT moiety (*m*/*z* 169.05 or 151.04) were also demonstrated in M2-23a. When matching the fragments of M2-30, a similar fragmentation pattern supported such a characterization, and diagnostic ions corresponding to the hydroxylated CA moiety (*m*/*z* of 195.03, 177.02, or 149.02) were also demonstrated in M2-23b.

##### Hydrated, Hydroxylated, and Acetylated Metabolites (+ H_2_O and +O and + C_2_H_2_O, +76 Da)

M2-31 [*m*/*z* 861.26459 (C_37_H_49_O_23_), eluted at 15.01 min] was 18 Da (+H_2_O) higher than M2-30, indicating that M2-31 was the hydrated product of M2-30. In the MS/MS spectrum of M2-31, the fragment ion at *m*/*z* 843.25 (C_37_H_47_O_22_) was formed by a loss of H_2_O from the quasi-molecular ion and showed a series of similar product ions of M2-30, indicating that the hydration site was at the α′, β′-d-double bond of the CA moiety with the presence of ions at *m*/*z* 137.02 (C_7_H_5_O_3_).

M2-25 [*m*/*z* 699.21606 (C_31_H_39_O_18_), eluted at 16.44 min] was 18 Da (+H_2_O) higher than M2-23 [*m*/*z* 681.20 (C_31_H_37_O_17_)], corresponding to the fragment ion at *m*/*z* 681.20 (C_31_H_37_O_16_) by a loss of H_2_O. A similar fragmentation pattern supported such a characterization, and diagnostic ions corresponding to the hydroxylated CA moiety (*m*/*z* of 195.03, 177.02, or 149.02) were also demonstrated in M2-25, indicating that M2-25 was the hydrated CA product of M2-23b with the presence of ion at *m*/*z* 137.02 (C_7_H_5_O_3_).

### Intestinal Microbial Metabolism Characterization of Four PhGs

**Figure 6:**
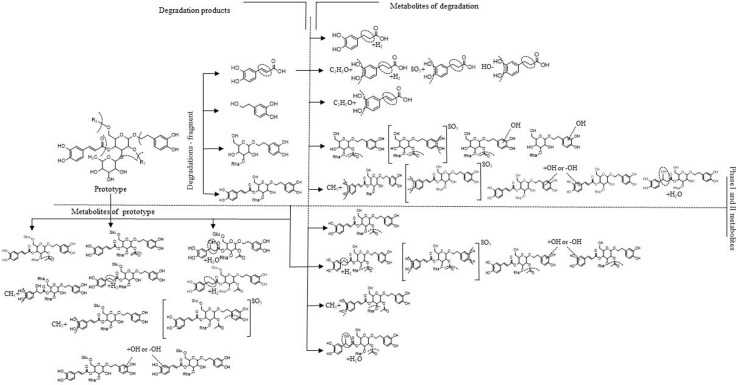
The main proposed metabolic pathways of PhG-related metabolites by rat intestinal bacteria.

Based on the above-identified metabolites, it was shown that PhGs go through extensive metabolism by intestinal microbiota *in vitro*, leading to low oral bioavailability. The main proposed metabolite pathways are shown in **Figure 6**, indicating that degradation was the main pathway, along with reduction, acetylation, sulfate conjugation, hydroxylation, methylation, dihydroxylation, and hydration. Particularly, the degradation pathways occurring in glycosidic or ester bonds could be simplified by reference on the mass fragmentation patterns.

Employing the effective data processing approach described above, the direct match and transformation-shifted fragments were rapidly identified and automatically annotated using the predicted fragmentation patterns and spectral annotation functions, thus facilitating elucidations on structures and localizations on metabolite sites. In the MS/MS spectra, our results suggested that the α′, β′-double bond and the phenolic hydroxyl group of the CA or HT moiety are the main metabolic sites, and the fragmentation patterns also have some regularities after being metabolized in the transformed moieties. Apart from the neutral losses and series of diagnostic ions corresponding to transformed CA or HT, other identical fragment ions were formed by the same fragmentation patterns as the parent compounds (see the section Metabolite Identification of four PhGs by Rat Intestinal Bacteria). A detailed summary of the information pertaining to metabolism is in **Table S2**. Using these fragmentation features, we rapidly and confidently identified more PhG-related metabolites or isomers. This study extended the current understanding of PhG metabolisms.

### Antioxidant Effect Evaluation Using the DPPH Assay *in vitro* and Structure–Antioxidant Activity Relationships of Typical PhGs and Related Metabolites

It is worth noting that PhGs have been shown to possess effective antioxidant activity. The degradation products of PhGs, especially CA, HT, and the derivatives, are also known potent antioxidants with many beneficial effects on human health (Selma et al., 2009; Peyrol et al., 2017; Alson et al., 2018; Shahidi and Yeo, 2018; Wu et al., 2018). The intestinal microbial metabolism characterizations provide valuable information to understand the efficacy and mechanism of PhG action and trace the potential therapeutic effects of metabolites. To better understand the mechanism of PhG action *in vitro*, SAR studies are warranted to identify the structural features essential for the biological activities and screen for more active components.

**Figure 7:**
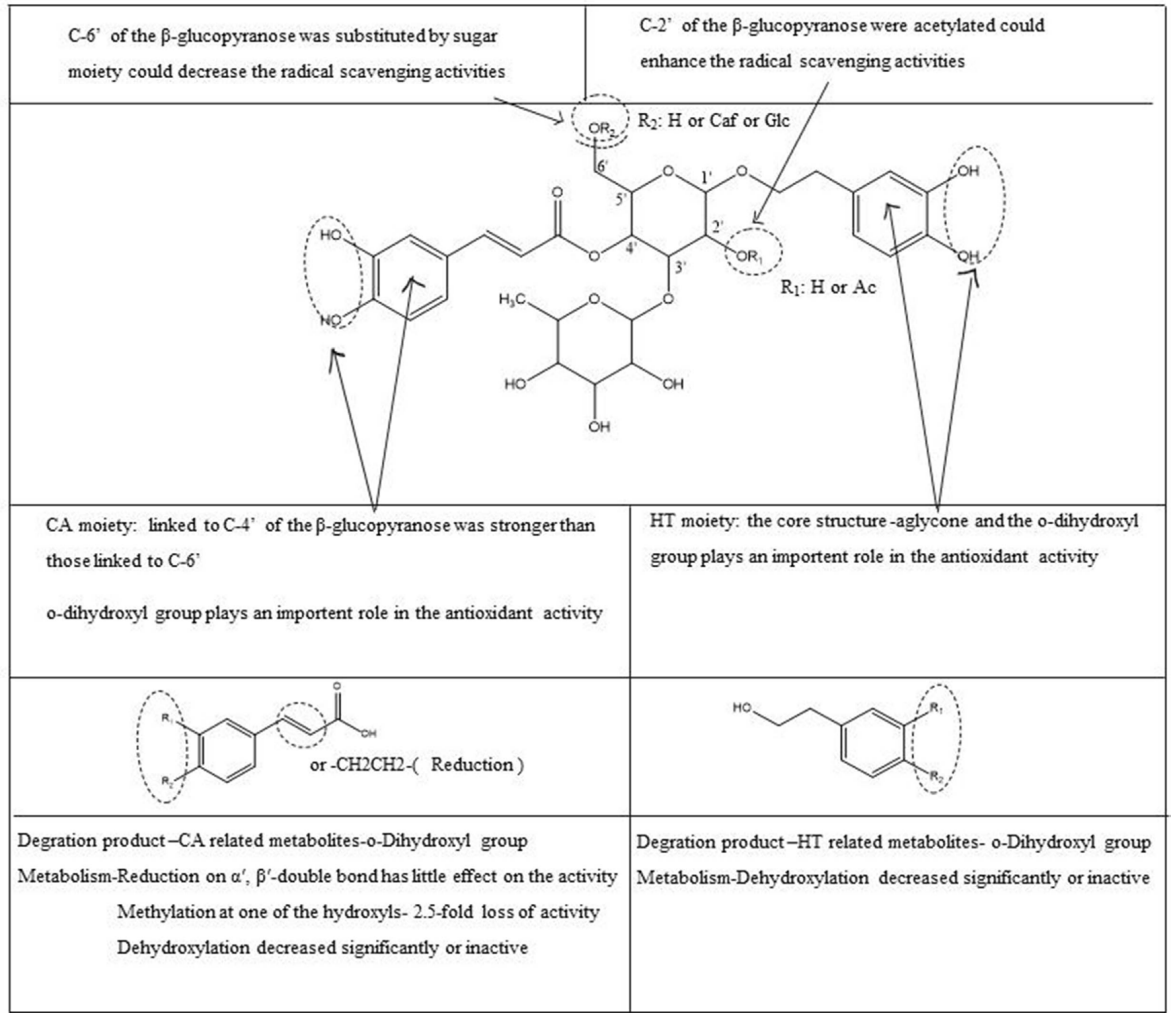
Summary of SAR study of PhGs and related metabolites.

In the present study, antioxidant activity evaluations on a) PhGs, b) CA-related metabolites, and c) HT-related metabolites were evaluated using the DPPH assay (**Table 1**), and some SARs were obtained (**Figure 7**). The DPPH radical scavenging activities of the tested compounds were expressed as an IC_50_ value, which is the effective compound concentration that resulted in 50% of scavenged radicals. Lower IC_50_ values indicate stronger antioxidant activities.

**Table 1:** Evaluation of PhGs, HT-related and CA-related metabolites by DPPH assay.

Class	Compounds	IC_50_ (µg/mL)
	2′-Acetylacteoside	1.155
	Acteoside	3.166
	Echinacoside	4.799
	Isoacteoside	3.235
PhGs	Poliumoside	3.613
	Tubuloside A	3.452
	Tubuloside B	2.469
	Osmanthuside B	11.103
	Saildroside	>100
	Caffeic acid	1.601
	3,4-Dihydroxybenzenepropanoic acid	1.556
	p-Hydroxycinnamic acid	>100
	m-Hydroxycinnamic acid	>100
	3-Hydroxyphenylpropionic acid	>100
CA related	3-(4-Hydroxyphenyl) propionic acid	>100
	Cinnamic acid	–
	Hydrocinnamic acid	–
	Ferulic acid	3.952
	Isoferulic acid	4.028
HT related	Hydroxytyrosol	1.312
	p-Tyrosol	>100

Based on the structures and IC_50_ values of these compounds, we theorize the following: 1) With respect to HT or CA and the related further metabolites, degradation products, such as HT, CA, and the reduction product of CA {3,4-dihydroxyphenylpropionic acid [dihydrocaffeic acid (DCA)]}, show the strongest radical scavenging activity (1.312 ± 0.06 to 1.601 ± 0.08 µg/mL) compared to trolox (VE) (2.638 ± 0.1 µg/mL), indicating that reduction on the α′, β′-double bond has little effect on the activity. Methylation at one of the hydroxyls resulted in an approximate 2.5-fold loss of activity among ferulic acid and isoferulic acid. In addition, because of dehydroxylation at one of the hydroxyls, the radical scavenging activity decreased significantly among p-tyrosol, m-hydroxycinnamic acid, p-hydroxycinnamic acid, 3-hydroxyphenylpropionic acid, and 3-(4-hydroxyphenyl) propionic acid (IC_50_ > 100 µg/mL). Cinnamic acid and hydrocinnamic acid are inactive (IC_50_ > 1,000 µg/mL), indicating that the ortho-dihydroxyphenyl structure in the HT and CA group is critical for the effects of DPPH-scavenging and antioxidants. 2) HT or CA glycosylated to PhGs reduce activity. Regarding PhGs, trisaccharide glycoside showed weaker activity than disaccharide glycoside, indicating that the sugar groups result in lower radical-scavenging activity due to steric hindrance, but the type of substituted sugar group has little effect on the activity. In addition, the radical scavenging activity significantly increased and the hydroxyl groups on the C-2′ of the β-glucopyranose were acetylated and the CA moiety linked to C-4′ of the central Glu was stronger than the moieties linked to C-6′, referring to 2′-acetylacteoside with the lowest IC_50_ (1.155 ± 0.05 µg/mL), followed by tubuloside B, acteoside, isoacteoside, tubuloside A, poliumoside, and echinacoside (2.469 ± 0.1 to 4.799 ± 0.2 μg/mL), and close to the positive control [trolox (VE); 2.638 ± 0.1 µg/mL], while osmanthuside B (11.103 ± 0.6 µg/mL) and saildroside (> 100 µg/mL) had only one hydroxyl group in the CA or HT group, exhibiting weaker activity compared to the tested PhGs and the other positive control [L-ascorbic acid (VC); 5.292 ± 0.5 µg/mL]. The SAR study may provide new insight into the development of lead compounds with enhanced pharmacologic activity; however, further studies are warranted to elucidate antioxidant activity *in vivo*, as well as the mechanism of action related to pharmacologic activity.

## Conclusions

In summary, systematic research on intestinal microbe-mediated metabolism and pharmacologic activities of four typical bioactive PhGs has been reported for the first time. The metabolite profiles using a rat intestinal bacteria incubation system, including a total of 26, 42, 42, and 36 metabolites, were characterized using UHPLC-Q-Exactive Orbitrap HRMS with automated data analysis software (Compound Discoverer). The accurate masses of precursor and fragment ions, automatic structural annotation, and transformation localization provided in this process showed more confidence in the metabolic characterization.

More metabolites were first identified in the current study, revealing that PhGs go through extensive metabolism, mainly involving deglycosylation, reduction, hydroxylation, hydration, methylation acetylation, and sulfate conjugation. The main metabolic sites were the α′, β′-double bond and phenolic hydroxyl group of the CA or HT moiety, and characteristic fragmentation patterns of PhGs and their metabolites were summarized in detail. Moreover, antioxidant activity evaluations focusing on both parent compounds and the metabolites were compared with the rapid DPPH-based assay *in vitro* and explored in SAR studies for the first time. Some metabolites still had potent antioxidant activity (superior DPPH radical scavenging capacities than VE and VC), which provides a scientific basis on elucidation of effective forms of PhGs with poor bioavailability by oral administration. In this review, we showed evidence of interactions with gut microbiota that PhG conversion into active and bioavailable metabolites, which help understand the dietary health effects of PhGs. Furthermore, SAR studies suggested that ortho-dihydroxyphenyl, C-2′-acetyl, and steric hindrance have an influence on the activity; thus, active metabolites formed by hydroxylation, deglycosylation, and C-2′ acetylation. Above all, it seemed that intestinal microbe-mediated metabolism plays an important role in regulating not only the pharmacokinetic but also the pharmacologic effects of PhGs. Therefore, the proposed integrative strategy was powerful for further exploration of the role of intestinal bacteria in the metabolism and mechanism of action underlying other natural products.

## Data Availability

The raw data supporting the conclusions of this manuscript will be made available by the authors, without undue reservation, to any qualified researcher.

## Author Contributions

YS and XW participated in research design. XW, XC, XL, MS, RX, and JC were responsible for performing the experiments. XW, YD, and YS performed data analysis. XW and YS contributed to the writing and editing of the manuscript. All authors have read and approved this article.

## Funding

This work was financially supported by National Natural Science Foundation of China (No.31570344) and the Chinese Academy of Medical Sciences (CAMS) Innovation Fund for Medical Sciences (CIFMS) (No. 2016-I2M-1-012).

## Conflict of Interest Statement

The authors declare that the research was conducted in the absence of any commercial or financial relationships that could be construed as a potential conflict of interest.

## Supplementary Materials

Table S1 Metabolite identification of four PhGs by rat intestinal bacteria.

Table S2 A detailed summary of the information on all metabolisms.
