# Supplementary material for: An Integrated Approach to Characterize Intestinal Metabolites of Four Phenylethanoid Glycosides and Intestinal Microbe-Mediated Antioxidant Activity Evaluation In Vitro Using UHPLC-Q-Exactive High-Resolution Mass Spectrometry and a 1,1-Diphenyl-2-picrylhydrazyl-Based Assay
Source: Front Pharmacol. 2019 Jul 25;10:826. doi: 10.3389/fphar.2019.00826 (PMC6669795; doi:10.3389/fphar.2019.00826)
Supplement: Table S1 — Metabolite identification of four PhGs by rat intestinal bacteria. [file Table_1.docx]

Supplementary data

Table S1 Metabolites identification of four PhGs by rats Intestinal bacteria

| Parents | No. | RT.  (min) | [M-H]^-^ m/z (Formula, ppm) | Fragment ions  m/z | Metabolite description |
| --- | --- | --- | --- | --- | --- |
| Poliumoside | M1 | 23.85 | 769.25592 (C_35_H_45_O_19_, -0.172) | 607.22437,461.16647,443.15613,315.10931,179.03455,161.02423,135.0450 | Parent |
|  | M1-1 | 6.62 | 153.05557 (C_8_H_9_O_3_, -0.964) | 123.04504 | HT |
|  | M1-2 | 7.97 | 341.08850 (C_15_H_17_O_9_, 2.037) | 179.03479,161.02426,135.04503 | M1-2Rha-HT |
|  | M1-3 | 9.94 | 607.22443 (C_26_H_39_O_16_, 0.118) | 461.16577,443.15759,315.10797,163.06136153.05556,123.04506 | M1-CA |
|  | M1-4 | 10.23 | 181.05061 (C_9_H_9_O_4_, -0.122) | 137.06068,119.05002 | 3, 4-Dihydroxybenzenepropionic acid |
|  | M1-5 | 12.31 | 179.03486 (C_9_H_7_O_4_, -0.681) | 135.04507 | CA |
|  | M1-6 | 14.15 | 803.26215 (C_35_H_47_O_21_, 0.770) | 785.24908,607.22339,461.16554,195.02960,177.01921,153.05531,149.02428,137.02428 | Hydroxylation and Hydration at CA moiety |
|  | M1-7a | 17.26 | 785.25067 (C_35_H_45_O_20_, -0.378) | 767.23621,633.20294,623.21808,179.03490,169.05009,161.02426,151.03989,135.04504 | Hydroxylation at HT moiety |
|  | M1-7b | 21.82 | 785.25232 (C_35_H_45_O_20_, 1.723) | 607.22437,195.02904,177.01915,153.05518,149.02422,135.04512,133.02942,123.04507 | Hydroxylation at CA moiety |
|  | M1-8 | 11.95 | 165.05547 (C_9_H_9_O_3_, -1.499) | 147.04503,119.05009,103.05515 | 3-Hydroxyphenylpropionic |
|  | M1-9 | 16.76 | 787.26459 (C_35_H_47_O_20_, -2.574) | 769.25562,607.22583,461.16635,179.03404,161.02426,153.05539,137.02426,135.04507 | Hydration at CA moiety |
|  | M1-10 | 24.24 | 801.28125 (C_36_H_49_O_20_, -1.269) | 769.25616,607.22449,461.16626,179.03491,175.03883,161.02423,153.05553,135.04507 | Methylation and Hydration at CA moiety |
|  | M1-11a | 22.77 | 849.21265 (C_35_H_45_O_22_S, -0.255) | 258.99170,215.00185,179.03485,161.02422,153.0547,135.04509,123.04492 | Sulfation at CA moiety |
|  | M1-11b | 12.27 | 849.21338 (C_35_H_45_O_22_S, 0.605) | 687.18073,233.01228,215.00174,179.03513,161.0242,135.04515 | Sulfation at HT moiety |
|  | M1-11c | 23.53 | 849.21289 (C_35_H_4_5O_22_S, 0.027) | 769.25610,607.22369,461.16687,179.03511,161.02426,153.05574,135.04514 | Sulfation |
|  | M1-12 | 23.70 | 771.27032 (C_35_H_47_O_19_, -1.792) | 607.22412,461.16818,443.15491,181.05028,153.05560,137.06065 | Reduction at CA moiety |
|  | M1-13a | 30.41 | 811.26685 (C_37_H_47_O_20_, 0.287) | 769.25610,607.22626,179.03500,161.02423,153.05536,135.04509,123.04518 | Acetylation |
|  | M1-13b | 24.73 | 811.26447 (C_37_H_47_O_20_, -2.646) | 195.06898,179.03609,161.02417,135.04538 | Acetylation at HT moiety |
|  | M1-13c | 29.52 | 811.26367 (C_37_H_47_O_20_, -3.632) | 769.25720,203.03485,179.03529,177.05577,161.02429, 159.04506, 135.04514 | Acetylation at CA moiety |
|  | M1-14 | 10.89 | 223.06085 (C_11_H_11_O_5_, -1.554) | 179.07108,163.03999,135.04495 | Reduction and acetylation of CA |
|  | M1-15 | 15.76 | 221.0453 (C_11_H_9_O_5_, -1.116) | 177.05554,149.06064 | Acetylation of CA |
|  | M1-16 | 17.84 | 801.2453 (C_35_H_45_O_21_, -0.726) | 783.23700,607.22302,193.01399,165.01907,153.05568,149.02435,137.02417,123.04507 | Dihydroxylation at CA moiety |
|  | M1-17 | 19.15 | 783.23444 (C_35_H_43_O_20_, -1.119) | 633.20557,621.20294,475.14514,179.03494,167.03468, 161.02428,149.02454,135.04507 | Hydroxylation and Dehydrogenation at HT moiety |
|  | M1-18 | 29.90 | 665.20905 (C_31_H_37_O_16_, 0.514) | 503.17719,461.16571,315.10788,179.03467161.02431,153.05554,135.04515,123.04499 | Acetylation of M1-Rha |
|  | M1-19 | 7.04 | 475.14523 (C_20_H_27_O_13_, -1.019) | 329.08719,167.03476,149.02422,123.04485 | M1-Rha-CA, Dehydrogenation and Hydroxylation at HT moiety |
|  | M1-20a | 23.14 | 623.19812 (C_29_H_35_O_15_, -0.038) | 461.16641,315.10852,179.03487,161.02423,153.05637,135.04509,123.04488 | M1-Rha or its isomers |
|  |  | 26.04 | 623.19830 (C_29_H_35_O_15_, 0.251) | 461.16580,179.03502,161.02431,153.05560,143.03465,135.04504,123.04510 |  |
|  | M1-20b | 22.22 | 623.19788 (C_29_H_35_O_15_, -0.423) | 461.16620,179.03487,161.02425,153.05505,143.03444,135.04504 |  |
|  |  | 25.14 | 623.19830 (C_29_H_35_O_15_, 0.251) | 461.16580,179.03502,161.02431,153.05560,143.03465,135.04504 |  |
|  | M1-21 | 8.86 | 487.14523 (_C21_H_27_O_13_, -0.994) | 179.03487,161.02423,135.04510 | M1-Rha-HT |
|  | M1-22 | 8.77 | 461.16632 (C_20_H_29_O_12_, -0.281) | 315.10794,163.06126,153.05527,143.03488 | M1-Rha-CA |
|  | M1-23a | 16.12 | 639.19434 (C_29_H_35_O_16_, 2.006) | 621.18268,487.14948,179.03474,161.02411,151.03941,135.04483,133.02948 | M1-Rha and Hydroxylation at HT moiety |
|  | M1-23b | 20.27 | 639.1914 (C_29_H_35_O_16_, -2.594) | 461.16583,177.01810,133.02927 | M1-Rha and Hydroxylation at CA moiety |
|  | M1-24 | 18.93 | 637.17773 (C_29_H_33_O_16,_ 0.505) | 475.14502,179.03488,161.02393,149.02377 | M1-Rha, Hydroxylation and Dehydrogenation  at HT moiety |
|  | M1-25 | 28.18 | 637.21503 (C_30_H_37_O_15_, 1.940) | 461.16571,193.05032,175.03983,153.05569 | M1-Rha and Methylation at CA moiety |
|  | M1-26 | 9.26 | 261.00671 (C_9_H_9_SO_7_, -2.822) | 181.04980,137.06049 | Reduction and Sulfation at CA |
| Echinacoside | M2 | 16.73 | 785.25031 (C_35_H_45_O_20_, -0.836) | 623.21887,477.16013,461.16519,315.10934,179.03468,161.02414,153.0554,135.0450 | Parent |
|  | M2-1 | 6.65 | 153.05560 (C_8_H_9_O_3_, -0.767) | 123.04511 | HT |
|  | M2-2 | 6.87 | 477.16132 (C_20_H_29_O_13_, -0.092) | 315.10825,179.05579,161.04524,153.05585,135.04518,123.04510,113.02435 | M2-Rha-CA |
|  | M2-3 | 8.10 | 461.16669 (C_20_H_29_O_12_, 0.522) | 315.10870,297.09808,161.04547,153.05545,143.03520,135.04518,113.02439 | M2-Glu-CA |
|  | M2-4 | 8.91 | 487.14587 (C_21_H_27_O_13_, 0.320) | 325.11417,179.03481,161.02420,135.04515 | M2-HT-Glu |
|  | M2-5 | 10.29 | 181.05060 (C_9_H_9_O_4_, -0.177) | 137.06075,119.05009 | 3, 4-Dihydroxybenzenepropionic acid |
|  | M2-6 | 12.33 | 179.03494(C_9_H_7_O_4_, -0.235) | 135.04517 | CA |
|  | M2-7 | 14.52 | 165.05563 (C_9_H_9_O_3_, -0.530) | 147.04503,119.05009,103.05515 | 3-Hydroxyphenylpropionic |
|  | M2-8 | 15.75 | 221.04541 (C_11_H_9_O_5_, -0.618) | 177.05565,149.06071 | Acetylation of CA |
|  | M2-9 | 10.92 | 223.06062 (C_11_H_11_O_5_, -2.586) | 179.07047,163.03983,135.04517 | Reduction and Acetylation of CA |
|  | M2-10 | 12.43 | 503.17758 (C_22_H_31_O_13_, 1.125) | 461.16638,315.10803,153.05576,135.04514 | Acetylation of M2-Glu-CA |
|  | M2-11 | 10.88 | 519.17218 (C_22_H_31_O_14_, 0.484) | 477.16125,315.10760,179.05595,153.05573 | Acetylation of M2-Rha-CA |
|  | M2-12 | 11.09 | 529.15594 (C_23_H_29_O_14_, -0.640) | 365.08722,179.03502,161.02437,135.04513 | Acetylation of M2-HT-Glu |
|  | M2-13 | 31.17 | 519.15039 (C_25_H_27_O_12_, -0.789) | 459.12973,297.09750,179.03456,161.02432 | Acetylation of M2-Rha-Glu |
|  | M2-14a | 26.62 | 607.20343 (C_29_H_35_O_14_, 0.331) | 445.17123,179.03467,161.02434,137.06079,133.02950,119.05238 | M2-Glu and Dehydroxylation at HT moiety |
|  | M2-14b | 27.22 | 607.20477 (C_29_H_35_O_14_, 2.538) | 461.16714,315.11072,163.03993,153.05571,145.02946,135.04547,123.04510,119.05015 | M2-Glu and Dehydroxylation at CA moiety |
|  | M2-15 | 23.14 | 623.19812 (C_29_H_35_O_15_, -0.038) | 461.16644,315.10968,179.03488,161.02431,153.05574,135.04517, 123.04490 | M2-Glu or isomers |
|  |  | 26.08 | 623.19812 (C_29_H_35_O_15_, -0.038) | 461.16644,315.10968,179.03488,161.02431,153.05574,135.04517,123.04490 |  |
|  | M2-16 | 28.20 | 637.21271 (C_30_H_37_O_15_, -1.700) | 461.16675,193.05037,179.03510,175.03983,161.02455, 153.05574,149.06099,135.04518 | M2-Glu and Methylation at CA moiety |
|  | M2-17a | 16.08 | 639.19293 (C_29_H_35_O_16_, -0.200) | 621.18152,487.14517,477.16235,179.03503,161.02441,151.04019,135.04514 | M2-Glu and Hydroxylation at HT moiety |
|  | M1-17b | 20.31 | 639.19324 (C_29_H_35_O_16_, 0.285) | 461.16693,177.01950,151.04076,133.02965 | M2-Glu and Hydroxylation at CA moiety |
|  | M2-18 | 19.03 | 637.17871 (C_29_H_33_O_16_, 2.043) | 487.14536,475.14691,329.08875,179.03503,167.03522,161.02480,149.02423,135.04543 | M2-Glu, Hydroxylation and Dehydrogenation at HT moiety |
|  | M2-19 | 23.61 | 665.22815 (C_28_H_41_O_18_, -2.537) | 623.21918,477.16190,461.16946,315.10843,179.05580,161.04512,153.05534,135.04512 | Acetylation of M2-CA |
|  | M2-20 | 31.82 | 665.20886 (C_31_H_37_O_16_, 0.228) | 623.20020,503.17523,461.16656,315.10886,179.03520,161.02435,153.05502,133.02951 | Acetylation of M2-Glu |
|  | M2-21 | 28.74 | 667.22518 (C_31_H_39_O_16_, 1.232) | 625.21478,503.17499,461.16544,315.10971,181.05075,163.04060,153.05588,137.06075 | Acetylation of M2-Glu and Reduction at CA moiety |
|  | M2-22 | 27.31 | 679.18593 (C_31_H_35_O_17_, -3.007) | 529.15527,517.15588,457.13254,305.06863,179.03491,161.02444,167.03444,149.02397 | Acetylation of M2-Glu, Hydroxylation and Dehydrogenation at HT moiety |
|  | M2-23a | 20.48 | 681.20483 (C_31_H_37_O_17_, 1.772) | 663.19312,621.18384,529.15546,519.17310179.03511,161.02434,151.04008,133.02981 | Acetylation of M2-Glu and Hydroxylation at HT moiety |
|  | M2-23b | 27.84 | 681.20380 (C_31_H_37_O_17_, 0.260) | 503.17651,461.16772,315.10806,195.03128,177.01923,153.05574,149.02432, 135.04498 | Acetylation of M2-Glu and Hydroxylation at CA moiety |
|  | M2-24 | 20.77 | 683.21918 (C_31_H_39_O_17_, -0.136) | 665.20892,503.17838,461.16620,315.10913,179.03491,161.02434,153.05557,137.02431 | Acetylation of M2-Glu and Hydration at CA moiety or its isomers |
|  |  | 22.59 | 683.22046 (C_31_H_39_O_17_, 1.738) | 665.20905,503.17737,461.16782,315.10773,179.03514,161.02435,153.05595,137.02432 |  |
|  | M2-25 | 16.44 | 699.21606 (C_31_H_39_O_18_, 2.678) | 681.20483,639.19189,503.17725,461.16611,315.10748,195.02946,177.01926,153.05544,149.02437,137.02438,123.04512 | Acetylation of M2-Glu, Hydroxylation and Hydration at CA moiety |
|  | M2-26a | 19.71 | 769.25481 (C_35_H_45_O_19_, -1.615) | 623.21686,163.04018,153.05511,145.02940,135.04469,123.04489 | Dehydroxylation at CA moiety |
|  | M2-26b | 25.01 | 769.25421 (C_35_H_45_O_19_, -2.395) | 607.22308,179.03471,161.02434,137.06117,135.04532,119.05056 | Dehydroxylation at HT moiety |
|  | M2-27 | 23.64 | 827.26147 (C_37_H_47_O_21_, -0.074) | 665.23022,623.21906,477.16153,461.16592,315.10922,179.03491,161.02431,153.05568 | Acetylation of M2 or its isomers |
|  |  | 26.27 | 827.26129 (C_37_H_47_O_21_, -0.292) | 665.22858,623.21912,477.16125,461.16626,315.10919,179.03482,161.02428,153.05559 |  |
|  | M2-28 | 17.92 | 845.27271 (C_37_H_49_O_22_, 0.726) | 827.26208,665.22894,623.22083,477.16269,315.10840,179.03517,161.02440,153.05573137.02437,133.02950,123.04519 | Acetylation of M2 and Hydration at CA moiety |
|  | M2-29a | 24.41 | 907.21930 (C_37_H_47_O_24_S, 1.052) | 665.23053,258.99161,215.00491,161.02431,153.05544,123.04492 | Acetylation of M2 and Sulfation at CA moiety |
|  | M2-29b | 20.84 | 907.21881 (C_37_H_47_O_24_S, 0.512) | 745.18762,233.01253,215.00191,179.03479,161.02437,135.04491 | Acetylation of M2 and Sulfation at HT moiety |
|  | M2-30 | 16.39 | 843.25507 (C_37_H_47_O_22_, -1.632) | 665.23157,647.22168,605.21100,195.02950,177.01936,153.05588,135.04547,123.04506 | Acetylation of M2 and Hydroxylation at CA moiety |
|  | M2-31 | 15.01 | 861.26459 (C_37_H_49_O_23_, -2.811) | 843.25226,665.23102,647.21814,605.20947,177.01930,153.05669,137.02434,123.04514, | Acetylation of M2, Hydroxylation and Hydration at CA moiety |
|  | M2-32 | 9.33 | 261.00720 (C_9_H_9_SO_7_, -0.945) | 181.05066,137.06100 | Reduction and Sulfation at CA |
|  | M2-33a | 13.06 | 801.24628 (C_35_H_45_O_21_, 0.498) | 639.21201,179.03468,169.05052,161.02425,151.04012,139.04010,135.04501 | Hydroxylation at HT moiety |
|  | M2-33b | 15.39 | 801.24567 (C_35_H_45_O_21_, -0.264) | 623.22028,477.16168,461.16589,195.02962,177.01953,153.05617,149.02435 | Hydroxylation at CA moiety |
|  | M2-34 | 16.25 | 787.26796 (C_35_H_47_O_20_, 1.706) | 623.21822,181.05036,153.05563,137.06069 | Reduction at CA moiety |
|  | M2-35 | 14.95 | 799.23041 (C_35_H_43_O_21_, 0.224) | 637.19879,475.14493,179.03468,167.03500,161.02423,149.02446,137.02390 | Hydroxylation and Dehydrogenation at HT moiety |
|  | M2-36 | 23.67 | 799.26758 (C_36_H_47_O_20_, 1.205) | 623.22125,477.16119,193.04957,179.03503,175.04060,161.02429,153.05537, 149.06061 | Methylation at CA moiety |
|  | M2-37 | 17.59 | 817.27893 (C_36_H_49_O_21_, 2.139) | 785.25134,623.22003,193.04974,179.03477,161.02441,153.05551,149.06059,135.04521 | Methylation and Hydration at CA moiety |
|  | M2-38 | 21.65 | 655.22577(C_30_H_39_O_16_, 2.155) | 623.19617,461.16882,193.05016,179.03525,161.02434, 153.05561,149.06092,135.04497 | M2-Glu, Methylation and Hydration at CA moiety |
|  | M2-39 | 13.81 | 817.24133 (C_35_H_45_O_22_, 0.653) | 623.21875,461.16498,193.01433,165.01903,153.05553,149.02437,135.04523,123.04506 | Dihydroxylation at CA moiety |
|  | M2-40 | 18.23 | 655.18976 (C_29_H_35_O_17_, 2.728) | 461.16632,315.11008,193.01425,165.01917,153.05527,149.02451,135.04518,123.04523 | M2-Glu and Dihydroxylation at CA moiety |
|  | M2-41 | 24.68 | 829.27435 (C_37_H_49_O_21_, -3.414) | 665.23004,623.22131,181.05034,153.05620 | Acetylation of M2 and Reduction at CA moiety |
|  | M2-42 | 18.53 | 841.24060 (C_37_H_45_O_22_, -0.233) | 679.21052,179.03471,167.03412,161.02432 | Acetylation of M2, Hydroxylation and Dehydrogenation at HT moiety |
| Tubuloside A | M3 | 23.54 | 827.26190 (C_37_H_47_O_21_, 0.446) | 665.22845,623.21820,477.16074,443.15625,179.03455,161.02431,153.05557,133.02953 | Parent |
|  | M3-1 | 21.37 | 477.14020 (C_23_H_25_O_11_, -0.073) | 315.10794,179.03502,161.02432,153.05612 | M3-Glu-Rha-Ac |
|  | M3-2 | 6.86 | 477.16122 (C_20_H_29_O_13_, -0.302) | 315.10944,179.05551,161.04549,153.05582 | M3-Rha-CA- Ac |
|  | M3-3 | 6.62 | 153.05563 (C_8_H_9_O_3_, -0.571) | 123.04511 | HT |
|  | M3-4 | 16.66 | 623.21899 (C_26_H_39_O_17_, -0.454) | 477.16098,461.16611,315.10825,221.06667,179.05600,161.04535,153.05562,135.04509 | M3-CA-Ac or its isomers |
|  |  | 20.31 | 623.21918 (C_26_H_39_O_17_, -0.149) | 477.15973,461.16498,315.10907,221.06622,179.05626,161.04469,153.05566,135.04512 |  |
|  | M3-5 | 23.12 | 623.19836 (C_29_H_35_O_15_, 0.347) | 461.16595,315.10941,179.03508,161.02434,153.05589,133.02953 | M3-Ac-Glu or its isomers |
|  |  | 26.06 | 623.19843 (C_29_H_35_O_15_, 0.460) | 461.16779,179.03474,161.02429,153.05510,133.02945,123.04520 |  |
|  | M3-6 | 8.04 | 461.16653 (C_20_H_29_O_12_, 0.175) | 315.10870,161.04541,153.05545,135.04514 | M3-Glu-CA-Ac |
|  | M3-7 | 8.07 | 315.10870 (C_14_H_19_O_8_, 0.505) | 179.05629,161.04536,153.05518,135.04517 | M3-Glu-Rha-CA-Ac |
|  | M3-8 | 10.36 | 181.05055(C_9_H_9_O_4_, -0.453) | 137.06070,119.05023 | 3, 4-Dihydroxybenzenepropionic acid |
|  | M3-9 | 12.34 | 179.03485(C_9_H_7_O_4_, -0.737) | 135.04507 | CA |
|  | M3-10 | 12.33 | 503.17633 (C_22_H_31_O_13_, -1.360) | 461.16620,315.10773,153.05624,135.04504 | M3-Glu-CA |
|  | M3-11 | 14.51 | 165.05554 (C_9_H_9_O_3_, -1.075) | 147.04506,119.05007,103.05521 | 3-Hydroxyphenylpropionic |
|  | M3-12 | 11.13 | 529.15613 (C_23_H_29_O_14_, -0.281) | 365.08688,179.03496,161.02431,135.04523 | M3-Glu-HT |
|  | M3-13a | 15.53 | 865.21069 (C_35_H_45_O_23_S, 3.362) | 785.24945,623.22021,215.00182,179.03488,161.02428,135.04495 | M3-Ac and Sulfation at HT moiety |
|  | M3-13b | 16.00 | 865.20624 (C_35_H_45_O_23_S, -1.781) | 785.24750,623.21948,179.03447,161.02428,153.05531,135.04520,123.04483 | Sulfation of M3-Ac |
|  | M3-13c | 12.93 | 865.20917 (C_35_H_45_O_23_S, 1.605) | 258.99103,215.00133,161.02426,135.04515 | M3-Ac and Sulfation at CA moiety |
|  | M3-14 | 15.88 | 221.04547 (C_11_H_9_O_5_, -0.347) | 177.05568,149.06070 | Acetylation of CA |
|  | M3-16 | 22.49 | 683.22046 (C_31_H_39_O_17_, 1.738) | 665.20807,503.17648,461.16702,179.03589,161.02441,137.02432,135.04509 | M3-Glu and Hydration at CA moiety |
|  | M3-17 | 20.26 | 681.20215 (C_31_H_37_O_17_, -2.162) | 663.19580,529.15442,519.17303,179.03516,161.02443,151.03978,135.04482 | M3-Glu and Hydroxylation at HT moiety |
|  | M3-18 | 20.36 | 785.25085 (C_35_H_45_O_20_, -0.149) | 623.21869,477.15973,315.10709,179.03493,161.02432,153.05586,135.04515,123.04504 | M3-Ac |
|  | M3-19a | 15.59 | 703.15411 (C_29_H_35_O_18_S, -1.206) | 541.12256,395.06573,377.05481,233.01123,215.00177,161.02405,135.04538 | M3-Ac-Glu and Sulfation at HT moiety |
|  | M3-19b | 19.21 | 703.15540 (C_29_H_35_O_18_S, 0.629) | 557.09980,258.99182,215.00185,179.03380,161.02415,135.04526 | M3-Ac-Glu and Sulfation at CA moiety |
|  | M3-19c | 21.39 | 703.15552 (C_29_H_35_O_18_S, 0.799) | 623.19934,461.16653,315.10100,179.03468,161.02432,153.05586,135.04518,123.04499 | Sulfation of M3-Ac-Glu |
|  | M3-20a | 16.09 | 639.19269 (C_29_H_35_O_16_, -0.576) | 621.18274,487.14566,179.03490,169.04996,161.02432,151.04004,139.04001,135.04506, | M3-Ac-Glu and Hydroxylation at HT moiety |
|  | M3-20b | 20.25 | 639.19336 (C_29_H_35_O_16_, 0.472) | 461.16815,315.10904,195.02975,177.01923,153.05560,151.03943,123.04475 | M3-Ac-Glu and Hydroxylation at CA moiety |
|  | M3-21 | 19.08 | 637.17761 (C_29_H_33_O_16_, 0.317) | 475.14569,329.08838,179.03487,167.03474,161.02428,149.02411,135.04521,123.04520 | M3-Ac-Glu, Hydroxylation and Dehydrogenation at HT moiety |
|  | M3-22 | 31.05 | 519.15063 (C_25_H_27_O_12_, -0.326) | 179.03474,161.02431,135.04523,133.02951 | M3-Glu-Rha |
|  | M3-23 | 32.62 | 649.21460 (C_31_H_37_O_15_, 1.242) | 503.17603,461.16840,163.04022,145.02937 | M3-Glu and Dehydroxylation at CA moiety |
|  | M3-24 | 12.51 | 657.20441 (C_29_H_37_O_17_, 1.198) | 639.19281,461.16656,443.15515,315.10815,195.02957,177.01907,153.05544,137.02432 | M3-Ac-Glu, Hydroxylation and Hydration at CA moiety |
|  | M3-25a | 26.58 | 607.20306 (C_29_H_35_O_14_, -0.278) | 445.17303,299.11288,179.03470,137.06023 | M3-Ac-Glu and Dehydroxylation at HT moiety |
|  | M3-25b | 29.20 | 607.20349 (C_29_H_35_O_14_, 0.430) | 461.16638,315.10815,163.03999,153.05557 | M3-Ac-Glu and Dehydroxylation at CA moiety |
|  | M3-26 | 18.45 | 841.23883 (C_37_H_45_O_22_, -2.337) | 179.03485,167.03392,161.02428,149.02422 | Hydroxylation and Dehydrogenation at HT moiety |
|  | M3-27 | 23.27 | 679.18854 (C_31_H_35_O_17_, 0.835) | 517.15350,475.14432,329.08868,179.03473,167.03473,161.02420,149.02432,135.04405 | M3-Glu, Hydroxylation and Dehydrogenation at HT moiety |
|  | M3-28a | 15.46 | 801.24721 (C_35_H_45_O_21_, 1.658) | 623.22131,477.16263,461.16470,195.02977,177.01923,153.05481,149.02417 | M3-Ac and Hydroxylation at CA moiety |
|  | M3-28b | 13.02 | 801.24579 (C_35_H_45_O_21_, -0.114) | 783.23657,639.19348,621.20758,179.03490,161.02434,151.04007,139.04015 | M3-Ac and Hydroxylation at HT moiety |
|  | M3-29 | 14.92 | 799.23041 (C_35_H_43_O_21_, 0.224) | 781.21936,649.20007,637.19708,179.03491,167.03479,161.02428,149.02431,137.02434 | M3-Ac, Hydroxylation and Dehydrogenation at HT moiety |
|  | M3-30 | 28.76 | 667.22581 (C_31_H_39_O_16_, 2.176) | 625.21436,461.16586,181.05052,163.04022 | M3-Glu and Reduction at CA moiety |
|  | M3-31 | 17.58 | 817.27802 (C_36_H_49_O_21_, 1.026) | 785.25134,623.21899,477.16080,315.10913, 175.03989,153.05600, 149.06063,135.04510 | M3-Ac, Methylation and Hydration at CA moiety |
|  | M3-32 | 15.21 | 655.18762 (C_29_H_35_O_17_, -0.538) | 461.16428,193.01387,165.01938,149.02437 | M3-Ac-Glu and Dihydroxylation at CA moiety |
|  | M3-33 | 15.54 | 641.20837 (C_29_H_37_O_16_, -0.527) | 623.19806,461.16641,315.10764,179.03452,161.02426,153.05586,137.02428,123.04495 | M3-Ac-Glu and Hydration at CA moiety |
|  | M3-34 | 21.67 | 655.22479 (C_30_H_39_O_16_, 0.659) | 623.19818,461.16589,315.10855,193.05028, 175.03883,153.05560,135.04520 | M3-Ac-Glu, Methylation and Hydration at CA moiety |
|  | M3-35 | 10.62 | 223.06035 (C_11_H_11_O_5_, -3.796) | 179.07127,163.03998,135.04507 | Reduction and Acetylation of CA |
|  | M3-36 | 9.33 | 261.00742 (C_9_H_9_SO_7_, -0.102) | 181.05066,137.06100 | Reduction and Sulfation at CA |
|  | M3-37 | 24.68 | 829.27435 (C_37_H_49_O_21_, -3.414) | 665.23004,623.22131,181.05034,153.05620 | Reduction at CA moiety |
|  | M3-38 | 16.54 | 843.25897 (C_37_H_47_O_22_, 2.993) | 825.24774,179.03442,161.02441,151.03979,135.04486,133.02931,123.04488 | Hydroxylation at HT moiety |
|  | M3-39 | 17.89 | 845.27061 (C_37_H_49_O_22_, -1.758) | 827.26221,665.22974,477.16187,179.03377,161.02435,153.05577,137.02434,135.04523 | Hydration at CA moiety |
|  | M3-40 | 14.17 | 803.26263 (C_35_H_47_O_21_, 1.368) | 785.25140,623.21851,179.03513,161.02425,153.05592,137.02419,135.04494,123.04484 | M3-Ac and Hydration at CA moiety |
|  | M3-41 | 13.73 | 817.23987 (C_35_H_45_O_22_, -1.133) | 193.01389,165.01874,149.02444,137.02446 | M3-Ac and Dihydroxylation at CA moiety |
|  | M3-42 | 5.21 | 477.16132 (C_20_H_29_O_13_, -0.092) | 459.15097,163.06171,161.04541,151.04002 | M3-Glu-CA-Ac and Hydroxylation at HT moiety |
| 2′-Acetylacteoside | M4 | 29.91 | 665.20874 (C_31_H_37_O_16_, 0.048) | 623.19769,503.17511,461.16574,315.10800,179.03490,161.02425,153.05545 | Parent |
|  | M4-1 | 6.58 | 153.05557 (C_8_H_9_O_3_, -0.964) | 123.04506 | HT |
|  | M4-2 | 9.36 | 357.11899 (C_16_H_21_O_9_, -0.323) | 315.10870,297.09775,153.05528,123.04504 | M4-Rha-CA |
|  | M4-3 | 11.25 | 583.13373 (C_22_H_31_O_16_S , -0.169) | 503.17551,461.16626,315.10867,153.05557 | Sulfation of M4-CA |
|  | M4-4 | 7.76 | 519.17120 (C_22_H_31_O_14_, -1.404) | 501.16168,477.16080,459.15118,313.09207,161.04529,151.03990,143.03461,123.04503 | M4-CA and Hydroxylation at HT moiety |
|  | M4-5 | 12.32 | 503.17694 (C_22_H_31_O_13_, -0.147) | 461.16635,443.15594,315.10831,161.04559,153.05579,143.03476,135.04512,123.04521 | M4-CA |
|  | M4-6 | 8.01 | 461.16635 (C_20_H_29_O_12_, -0.216) | 315.10889,161.04547,153.05553,143.03508 | M4-CA-Ac |
|  | M4-7 | 25.13 | 461.14545 (C_23_H_25_O_10_, 0.282) | 179.03452,161.02426,135.04477,119.04980 | M4-Ac-Rha and Dehydroxylation at HT moiety |
|  | M4-8 | 11.08 | 529.15521 (C_23_H_29_O_14_, -2.020) | 365.08902,179.03491,161.02434,135.04514 | M4-HT |
|  | M4-9 | 10.30 | 181.05052 (C_9_H_9_O_4_, -0.619) | 137.06070,119.05016 | 3, 4-Dihydroxybenzenepropionic acid |
|  | M4-10 | 12.18 | 571.16663 (C_25_H_31_O_15_, -0.373) | 529.15564,409.13528,367.12399,221.06668,179.03539,161.02429,143.03453,135.04510 | Acetylation of M4-HT |
|  | M4-11 | 12.24 | 445.17145 (C_20_H_29_O_11_, -0.191) | 299.11328,179.05589,161.04543,143.03471,137.06076,119.05012 | M4-CA–Ac and Dehydroxylation at HT moiety |
|  | M4-12 | 12.34 | 179.03491 (C_9_H_7_O_4_, -0.402) | 135.04510 | CA |
|  | M4-13 | 10.13 | 517.15552 (C_22_H_29_O_14_, -1.467) | 167.03496,163.06088,149.02428,121.02937 | M4-CA, Hydroxylation and Dehydrogenation at HT moiety |
|  | M4-14 | 14.49 | 165.05554 (C_9_H_9_O_3_, -1.075) | 147.04506,119.05008,103.05520 | 3-hydroxyphenylpropionic |
|  | M4-15 | 16.42 | 699.21368 ( C_31_H_39_O_18_, -0.726) | 681.20508,639.19214,503.17749,461.16684,315.10931,195.02988,177.01915,153.05482,149.02420,137.02429,123.04504 | Hydroxylation and Hydration at CA moiety |
|  | M4-16 | 15.41 | 545.18732 (C_24_H_33_O_14_, -0.475) | 503.17691,461.16705,443.15656,357.11972,315.10773,161.04500,153.05557,135.04509 | Acetylation of M4-CA |
|  | M4-17 | 31.11 | 519.15039 (C_25_H_27_O_12_, -0.789) | 477.14072,357.11856,297.09802,179.03476,161.02426,133.02945,123.04505 | M4-Rha |
|  | M4-18 | 22.49 | 683.21822 (C_31_H_39_O_17_, -1.541) | 665.20892,503.17740,461.17050,315.10889,179.03503,161.02429,153.05573,137.02428 | Hydration at CA moiety |
|  | M4-19 | 21.35 | 703.15723 (C_29_H_35_O_18_S, 3.231) | 623.20294,461.16464,161.02412,135.04445 | Sulfation of M4-Ac |
|  | M4-20 | 21.24 | 477.13998 (C_23_H_25_O_11_, -0.534) | 179.03423,161.02423,135.04515 | M4-Rha-Ac |
|  | M4-21a | 20.39 | 681.20300 (C_31_H_37_O_17_, -0.914) | 663.19257,529.15686,519.17255,365.08813,179.03471,161.02432,151.04001 | Hydroxylation at HT moiety |
|  | M4-21b | 24.14 | 681.20349 (_C31_H_37_O_17_, -0.195) | 503.17859,461.16492,315.10828,195.02995,177.01918,153.05559,149.02428,123.04495 | Hydroxylation at CA moiety |
|  | M4-22 | 23.06 | 623.19806 (C_29_H_35_O_15_, -0.134) | 461.16623,315.10788,179.03484,161.02429,153.05559,135.04509,123.04519 | M4-Ac or isomers |
|  |  | 26.11 | 623.19800 (C_29_H_35_O_15_, -0.230) | 461.16580,315.10849,179.03502,161.02431,153.05560,135.04504,123.04510 |  |
|  | M4-23a | 26.56 | 607.20367 (C_29_H_35_O_14_, 0.727) | 461.16653,315.10843,163.04005,153.05545,145.02940,135.04518,123.04512,117.03452 | M4-Ac and dehydroxylation at CA moiety |
|  | M4-23b | 29.17 | 607.20294 (C_29_H_35_O_14_, -0.476) | 445.17090,299.11331,179.03490,161.02428,137.05974,135.04478,119.05000 | M4-Ac and dehydroxylation at HT moiety |
|  | M4-24a | 22.44 | 745.16479 (C_31_H_37_O_19_S, -0.983) | 599.10760,258.99158,240.98122,215.00183,179.03508,161.02434,153.05515,123.04510 | Sulfation at CA moiety |
|  | M4-24b | 27.76 | 745.16559 (C_31_H_37_O_19_S, 0.090) | 665.20947,461.16544,315.10837,179.03499,161.02431,153.05600,123.04487 | Sulfation of M4 |
|  | M4-24c | 16.69 | 745.16425 (C_31_H_37_O_19_S, -1.708) | 233.01257,215.00200,179.03416,161.02422 | Sulfation at HT moiety |
|  | M4-25 | 33.57 | 649.21411 (C_31_H_37_O_15_, 0.487) | 607.20178,503.17712,461.16641,315.10745,163.04004,153.05539,145.02939,135.04507 | Dehydroxylation at CA moiety |
|  | M4-26 | 34.32 | 707.21887 (C_33_H_39_O_17_, -0.570) | 665.21082,503.17676,461.16626,315.10858,179.03468,161.02423,153.05568,123.04491 | Acetylation of M4 |
|  | M4-27 | 23.24 | 679.18811 (C_31_H_35_O_17_, 0.202) | 529.15582,517.15533,475.14557,329.08743,179.03525,167.03476,161.02432,149.02432 | Hydroxylation and Dehydrogenation at HT moiety |
|  | M4-28 | 9.31 | 261.00726 (C_9_H_9_O_7_S, -0.715) | 181.05057,137.06068 | Reduction and Sulfation of CA |
|  | M4-29a | 16.16 | 639.19312 (C_29_H_35_O_16_, 0.097) | 487.14398,179.03479,161.02426,151.03989 | M4–Ac and Hydroxylation at HT moiety |
|  | M4-29b | 20.24 | 639.19397 (C_29_H_35_O_16_, 1.427) | 461.16510,177.01918,149.02364,133.02937 | M4–Ac and Hydroxylation at CA moiety |
|  | M4-30 | 15.71 | 221.04539 (C_11_H_9_O_5_, -0.709) | 177.05554,149.06062 | Acetylation of CA |
|  | M4-31 | 10.91 | 223.06033 (C_11_H_11_O_5_, -3.886) | 179.07129,163.03998,135.04509 | Reduction and Acetylation of CA |
|  | M4-32 | 19.04 | 697.19904 (C_31_H_37_O_18_, 0.721) | 503.17630,193.01443,165.01932,149.02440,137.02426,123.04496 | Dihydroxylation at CA moiety |
|  | M4-33 | 28.72 | 667.22390 (C_31_H_39_O_16_, -0.687) | 625.21200,461.16663,181.05037,153.05580 | Reduction at CA moiety |
|  | M4-34 | 33.02 | 679.22626 (C_32_H_39_O_16_, 2.800) | 637.21393,503.17764,461.16559,315.10834,193.04997,175.03996, 153.05580,149.05989 | Methylation at CA moiety |
|  | M4-35 | 28.25 | 637.21411 (C_30_H_37_O_15_, 0.497) | 461.16623,315.10938,193.05037,175.03992,153.05577,149.06065,135.04524,123.04498 | M4–Ac and Methylation at CA moiety |
|  | M4-36 | 18.99 | 637.17780 (C_29_H_33_O_16_, 0.615) | 487.14520,475.14557,179.03484,167.03453,161.02473,149.02411,135.04500 | M4–Ac, Hydroxylation and Dehydrogenation at HT moiety |

Table S2 A detailed summary of the informations on all metabolisms

| Metabolite sites | Transformations | Formula change | Delta Mass | Fragmentation Features | |
| --- | --- | --- | --- | --- | --- |
|  |  |  |  | Neutral Loss | Diagnostic ions ( m/z ) |
| HT | - | - | - | C_8_H_8_O_2_ (136 Da) | 153.06, 135.04 and 123.04 |
|  | Hydroxylation | + O | + 16 Da | C_8_H_8_O_3_ (152 Da) | 169.05 and 151.04 |
|  | Hydroxylation & Dehydrogenation | + O- H_2_ | + 14 Da | C_8_H_6_O_3_ (150 Da) | 167.03 and 149.02 |
|  | Sulfation | + SO_3_ | + 80 Da | SO_3_ (80 Da) | 233.01, 215.00 and 153.06 |
|  | Dehydroxylation | - O | - 16 Da | - | 137.06 and 119.05 |
|  | Acetylation | + C_2_H_2_O | + 42 Da | C_2_H_2_O (42 Da) | 195.07 and 153.06 |
| CA | - | - | - | C_9_H_6_O_3_ (162 Da) | 179.03, 161.02 and 135.04 |
|  | Hydroxylation | + O | + 16 Da | C_9_H_6_O_4_ (178 Da) | 195.03, 177.02 and 149.02 |
|  | Reduction | + H_2_ | + 2 Da | C_9_H_8_O_3_ (164 Da) | 181.05, 163.04 and 137.06 |
|  | Sulfation | + SO_3_ | + 80 Da | SO_3_ (80 Da) | 258.99, 215.00 and 179.03 |
|  | Dehydroxylation | - O | - 16 Da | C_9_H_6_O_2_ (146 Da) | 163.04, 145.03 and 119.05 |
|  | Acetylation | + C_2_H_2_O | + 42 Da | C_2_H_2_O (42 Da) | 203.03, 179.03 and 177.06 |
|  | Hydration | + H_2_O | + 18 Da | H_2_O (18 Da) | 137.02 |
|  | Dihydroxylation | + 2O | + 32 Da | C_9_H_6_O_5_ (194 Da) | 193.01, 165.02 and 149.02 |
|  | Methylation | +CH_2_ | + 14 Da | C_10_H_8_O_3_ (176 Da) | 193.05, 175.04 and 149.06 |
